# Untargeted Metabolomics for Profiling of Cascara, Senna, Rhubarb, and Frangula Metabolites

**DOI:** 10.3390/metabo15120779

**Published:** 2025-12-03

**Authors:** Paola Nezi, Alessia Lucia Prete, Filippo Costanti, Vittoria Cicaloni, Mattia Cicogni, Laura Tinti, Laura Salvini, Monica Bianchini

**Affiliations:** 1 Fondazione Toscana Life Sciences, Via Fiorentina 1, 53100 Siena, Italy; v.cicaloni@toscanalifesciences.org (V.C.); m.cicogni@toscanalifesciences.org (M.C.); l.tinti@toscanalifesciences.org (L.T.); l.salvini@toscanalifesciences.org (L.S.); 2 Department of Information Engineering and Mathematics, University of Siena, Via Roma, 56, 53100 Siena, Italy; filippo.costanti@unisi.it (F.C.); monica.bianchini@unisi.it (M.B.)

**Keywords:** untargeted metabolomics, hydroxyanthracene derivatives, medicinal plants, secondary metabolites

## Abstract

Background/Objectives: Natural products containing hydroxyanthracene derivatives (HADs) such as Cascara (*Rhamnus purshiana*), Frangula (*Rhamnus frangula*), Rhubarb (*Rheum palmatum*), and Senna (*Cassia angustifolia*) have long been used for their laxative properties, but also raise safety concerns due to reported genotoxic and carcinogenic potential. Most studies have focused on quantifying HADs, whereas the broader secondary metabolite landscape of these herbal drugs remains underexplored. We aimed to generate an untargeted metabolomic fingerprint of these four species and to explore their chemical diversity using AI-based structural classification. Methods: Four commercial botanical raw materials were extracted with 60% methanol and analysed by UPLC–HRMS/MS in positive and negative ion modes. Features were processed in Compound Discoverer and annotated by accurate mass and MS/MS matching against spectral databases, then assigned to structural classes using a graph neural network classifier. Multivariate analyses (PCA, HCA) were used to compare metabolic patterns across species. Results: In total, 93, 83, 83 and 51 metabolites were annotated in cascara, frangula, rhubarb, and senna, respectively, spanning flavonoids, anthraquinones, phenylpropanoids and other classes. Only four flavonoids were shared by all species, indicating marked biochemical divergence. Several putatively species-enriched features were observed, including pavine in cascara and frangula, vicenin-2 in senna, and piceatannol in rhubarb. Senna displayed the most distinct metabolic profile, whereas cascara and frangula clustered closely. Conclusions: This work provides a chemistry-centred metabolomic fingerprint of four HAD-containing herbal drugs using graph-based neural networks for natural product classification, supporting future studies on the pharmacological potential, bioavailability and safety of their metabolites.

## 1. Introduction

Natural products play an important role in disease treatment and still represent the core of traditional medicine systems in several countries [1]. Among these are hydroxyanthracenes (HADs), a class of aromatic organic compounds widely present in nature and particularly in many medicinal plants, including *Cassia angustifolia* (Senna), *Rheum palmatum* (Rhubarb), *Rhamnus frangula* (Frangula), and *Rhamnus purshiana* (Cascara) [2].

HADs are secondary metabolites characterized by a 9,10-dioxoanthracene core, often substituted with one or more hydroxyl groups and occurring either as free aglycones or as glycosides conjugated to sugar moieties [3,4]. More than 700 natural HADs have been reported, with over 200 identified in flowering plants and the remainder in lichens and fungi [2]. IHADs are distributed in different organs (rhizomes, roots, bark, leaves, fruits) and are typically stored as glycosides, which facilitates their accumulation and modulates their bioactivation in the gastrointestinal tract [4]. Representative examples include Emodin, Aloe-emodin, and Rhein, which are among the main anthraquinone constituents of Rhubarb, Senna, Frangula, and Cascara [5,6].

HADs are known for their pharmacological properties, particularly laxative and digestive activities [6,7], and have been used for decades in numerous pharmaceutical formulations and dietary supplements. However, these compounds are not devoid of toxicity [8]. The genotoxicity of HADs derivatives has been evaluated in numerous in vitro and in vivo studies identified from the public literature. In particular, some epidemiological studies showed an increased risk of colorectal cancer [9,10,11,12]. For this reason, the European Food Safety Authority (EFSA) has re-evaluated the safety of the use of medicinal plants containing HADs in food supplements [13,14], concluding that they should be considered genotoxic and carcinogenic until proven otherwise. This assessment highlights the need for a more in-depth investigations of medicinal plants containing HADs for the whole spectrum of secondary metabolites of Senna, Rhubarb, Frangula, and Cascara.

The chemical and pharmacological properties of the plants *Rhamnus frangula* (Frangula), *Rhamnus purshiana* (Cascara), *Rheum palmatum* (Rhubarb), and *Cassia angustifolia* (Senna) have been the subject of several investigations. Many studies have already focused on HADs, but comparatively less is known about the broader spectrum of secondary metabolites present in these therapeutic plants. For instance, a recent study [15] combined qualitative–quantitative characterization of HADs in commercial preparations of Senna, Rhubarb, Cascara, and Frangula with cytotoxicity assays and shotgun proteomics in an intestinal cell model, comparing the effects of single HAD molecules with those of whole plant extracts. Together with the broader in vitro and in vivo literature and regulatory evaluations [12,13,16], this work highlights the complexity of HAD-containing products. However, a comprehensive, chemistry-centred description of the overall metabolite landscape of these herbal drugs is still lacking.

In the state of the art, the literature reports different studies on Cascara establishing anthraquinone glycosides as the active constituents of the bark [17]. Additional studies have focused on determining the presence of HADs using methods such as liquid chromatography combined with mass spectrometry [18,19]. While HADs in Frangula and Cascara, belonging to family Rhamnaceae, have been closely quantified and studied, other secondary metabolites have not been explored in as much detail [20,21].

Regarding Rhubarb, specific studies have primarily focused on quantifying HADs and phenolic compounds [22,23,24]. Specifically, rhein has been identified as the metabolite responsible for the toxicity of anthraquinones [16].

Conversely, numerous studies on Senna have concentrated on identifying the generated metabolites without being limited to HADs, in particular those with antibacterial properties [25]. The diversity of bioactive compounds has been revealed in different studies, characterizing and quantifying polyphenols and other phytochemicals [26,27,28].

Despite these efforts, no study has yet provided a comprehensive metabolic profiling of these four species. Untargeted metabolomics offers a powerful means to achieve this, enabling the detection and annotation of a wide diversity of metabolites and shedding light on their biological roles and potential health impacts.

In this context, the present work was designed as a complementary chemistry-centred investigation. Rather than re-evaluating bioavailability and safety, which have been specifically addressed in previous studies such as [15], our primary aim is to provide an untargeted UPLC–MS/MS metabolomic fingerprint of Cascara, Senna, Frangula, and Rhubarb and to explore their chemical diversity using AI-based classification approaches.

In addition to experimental metabolomics, recent advances in artificial intelligence have provided new opportunities for the structural classification of natural products. Tools such as NPClassifier [29] have demonstrated the feasibility of applying deep learning to metabolite categorization, although with limitations in capturing structurally diverse or less represented scaffolds. More recently, graph-based neural networks have shown superior performance in modeling molecular topology and enhancing classification accuracy, as described in [30]. By integrating such approaches, our study not only provides an untargeted metabolic profiling of anthraquinone-rich plants but also leverages state-of-the-art computational methods to achieve a higher-resolution view of their chemical diversity.

## 2. Materials and Methods

The four samples were kindly supplied by different companies. Botanical samples consisted of *Rheum palmatum* (Rhubarb) (root), *Cassia angustifolia* (Senna) (leaves), *Rhamnus purshiana* (Cascara) (bark), and *Rhamnus frangula* (Frangula) (bark). All materials were obtained as semi-processed dried plant organs in milled form. Samples were stored at 25 °C under controlled humidity in an ISO 9001:2015-certified facility (certificate Q/1765/24) [31], ensuring standardized workflows, traceability, and quality control in line with the storage requirements for semi-processed herbal materials used in food supplement manufacturing. Each powdered plant material (100 mg) was extracted by sonication for 20 min in 10 mL of 60% methanol (Merck Group (Darmstadt, Germany)). This hydroalcoholic mixture was selected on the basis of preliminary tests comparing methanol–water and ethanol–water systems (100%, 80%, 60%), which indicated that 60% methanol provided the best compromise between chromatographic signal intensity and metabolite coverage under the adopted LC–HRMS conditions. After centrifugation at 13,000 rpm for 10 min, the supernatant was collected, filtered through a 0.22 µm membrane, and injected directly into the UPLC–Q Exactive Plus system without dilution. All analyses for each sample were performed in technical triplicate. No biological replicates were included, as the study focused on metabolomic characterization of distinct herbal drugs that are used as sources of anthraquinone-containing ingredients in commercial laxative formulations.

The metabolic profiles of the powdered plant samples were analyzed using an Ultimate 3000 UPLC system (Thermo Fisher Scientific (Waltham, MA, USA)) coupled with a Q-Exactive Plus Hybrid Quadrupole–Orbitrap™ high-resolution mass spectrometer (Thermo Fisher Scientific). Data were acquired in both positive and negative electrospray modes over a scan range of *m*/*z* 200–2000. Operating parameters were as follows: spray voltage 3.5 kV (positive mode) and 3.0 kV (negative mode); sheath gas = 20 a.u.; auxiliary gas = 5 a.u.; capillary temperature = 320 °C; and resolution = 35,000. Acquisition was performed in Full MS/dd-MS^2^ (Top N) mode, selecting and fragmenting precursor ions according to intensity. MS^2^ spectra were generated using higher-energy collisional dissociation (HCD) at 30 a.u., with a mass accuracy threshold of 5 ppm. Chromatographic separation employed an Acquity UPLC BEH C18 column (2.1 mm × 150 mm, 1.7 µm; Waters (Milford, MA, USA)). The mobile phases were (A) water with 0.1% formic acid and (B) acetonitrile with 0.1% formic acid (Merck Group). A linear gradient was applied starting at 2% B (1 min hold), increasing to 100% B over 50 min, maintained for 2 min, then re-equilibrated to the initial conditions. The flow rate was 0.2 mL $\;\min^{-1}$, the injection volume was 10 µL, and the column temperature was maintained at 35 °C. Raw LC–MS/MS data were processed using Compound Discoverer 3.3 (Thermo Fisher Scientific). Feature detection and alignment were performed with default settings, except for a retention-time tolerance of 0.2 min and a mass tolerance of 10 ppm. Blank solvent runs were acquired under identical conditions to identify and exclude background peaks originating from the matrix or solvent, thereby enhancing annotation reliability.

### 2.1. Feature Extraction and Metabolite Annotation

Metabolite features were extracted and processed using Thermo Fisher’s Compound Discoverer (CD) software (v3.3). The workflow included automated feature detection, chromatographic alignment, background subtraction, isotope/adduct grouping, and compound annotation. For each detected feature, CD returned (when available) the compound name, molecular formula, precursor *m*/*z*, calculated molecular weight, retention time (RT), maximum peak area, ionization mode (ESI positive or negative), and MS/MS-based annotation obtained through matching against the spectral and structural databases integrated into the platform (mzCloud, mzVault, ChemSpider, Mass List, Metabolika). The full feature tables exported from CD containing these parameters for all detected compounds are provided as Supplementary Materials. In line with the Metabolomics Standards Initiative (MSI), metabolites confirmed with authentic reference standards and MS/MS fragmentation matching (e.g., the main hydroxyanthracene derivatives identified in the different samples) are classified as MSI Level 1, features annotated on the basis of accurate mass and MS/MS spectral similarity to database entries are classified as MSI Level 2, and unannotated or partially characterized features are assigned to MSI Levels 3/4. The MSI confidence level associated with each feature is explicitly indicated in the tables provided in the Supplementary Materials. As such, non–standard-confirmed metabolites discussed in the main text should be regarded as putative annotations (MSI Level 2) pending further validation with authentic standards.

### 2.2. Metabolite Screening Process

- Database Confirmation: Compounds showing full or partial correspondence in at least one of five reference databases (*m*/*z* Cloud, *m*/*z* Vault, Metabolika, ChemSpider, or Mass List) were included.
- Mass Accuracy: Deviation within ±3 ppm from theoretical *m*/*z*.
- RT (Retention Time): Compounds eluting between 5 and 50 min were selected, although the range could be extended (0–120 min) to accommodate specific analytical requirements.
- Peak Area: Features with areas below 1.0 × $10^{-5}$ were excluded in order to minimize low-intensity background signals.
- ${\mathrm{MS}}^{2}$ Availability: Only compounds with corresponding ${\mathrm{MS}}^{2}$ spectra were retained for annotation.

The resulting dataset was cross-checked against the published literature to verify compound identities and contextualize the detected metabolites within known phytochemical profiles.

### 2.3. Classification of Natural Products

For the structural classification of metabolites, we employed Graph Isomorphism Networks (GINs), following the framework described in [30]. GINs were selected due to their ability to capture molecular graph topology with high fidelity and to improve predictive performance in natural product classification tasks.

Each metabolite was converted from its SMILES notation into a graph representation, where atoms were encoded as nodes with associated features (atom type, degree, hybridization state, formal charge, aromaticity) and bonds were encoded as edges with features describing bond type and conjugation. These molecular graphs were then processed by GINs specifically trained for each classification level (pathway, superclass, and class), as reported in [30]. Technical details of the network architectures and training procedures—including layer composition, activation functions, optimization strategy, and hyperparameters—are provided in Table A1. Model training and validation were performed on curated datasets of annotated natural products using stratified 10-fold cross-validation to ensure robustness. Performance was assessed based on macro-averaged F1 score and accuracy.

The trained models were subsequently applied to the metabolite dataset generated by Compound Discoverer 3.3. The predicted class assignments were merged with experimental annotations, yielding a detailed structural classification of the identified compounds and enabling a more precise comparison of the metabolite composition across the investigated species.

### 2.4. Statistical Analysis

Intersections among metabolite lists were computed using the web-based tool available at the Bioinformatics & Evolutionary Genomics platform (https://bioinformatics.psb.ugent.be/webtools/Venn/ (accessed on 15 May 2025)). The application provided both textual and graphical outputs, identifying shared and unique metabolites across the compared datasets.

The processed data matrix was imported into MetaboAnalyst 6.0 6.0 [32] for multivariate analysis. Exploratory principal component analysis (PCA) was performed to visualize interspecies variation and clustering patterns, while hierarchical cluster analysis (HCA) based on Euclidean distance was used to construct a dendrogram illustrating metabolic relationships among the investigated samples after appropriate data filtering, normalization and scaling. No formal univariate or supervised hypothesis testing (e.g., ANOVA, *t*-tests, OPLS-DA) was performed; therefore, all comparisons of metabolite abundances are exploratory and descriptive. Consequently, no p-values were calculated and no false discovery rate (FDR) or multiple-testing corrections were applied.

Relative abundances of annotated metabolites were visualized through customized heatmaps generated via a dedicated Python script employing Pandas (v2.1.4), Matplotlib (v3.8), and Seaborn (v0.13).

## 3. Results

In this work, the dried materials of Cascara, Senna, Frangula, and Rhubarb were investigated to describe their non-volatile profiles through UPLC–MS/MS for the first time. These species are traditionally employed for their laxative properties [33], and their therapeutic relevance has sustained longstanding interest in their chemical composition. As such, a comprehensive characterization of their secondary metabolites is essential both to explain their pharmacological activity and to evaluate potential safety concerns associated with their use.

To complement proteomic findings, an untargeted metabolomic profiling was performed on representative methanolic extracts of Cascara, Frangula, Rhubarb, and Senna. Both positive and negative ionization modes were applied to obtain a comprehensive overview of the metabolite composition. The approach enabled the annotation of 93 metabolites in Cascara, 83 in Frangula, 83 in Rhubarb, and 51 in Senna. Complete compound lists are reported in Appendix A Table A2, Table A5, Table A8 and Table A11).

Given the structural diversity of natural products, a hierarchical classification framework is typically adopted to provide consistent annotation, organizing metabolites into three levels: pathway, superclass, and class [29]. Pathways reflect the major biosynthetic origins, superclasses capture broad chemical categories, and classes resolve scaffold-level diversity within each superclass. This organization enables both global metabolome profiling and detailed analyses of families of biological relevance. To characterize the metabolic diversity of the four species, the identified compounds were classified with the GIN network; results are reported at the superclass level, which offers a good balance between interpretability and comparability with previous studies.

Expert chemists verified the assignments at the superclass level. For Cascara, expert review confirmed 90/93 assignments (96.8% concordance), with only three discordant cases reported in Table A4. For Frangula, 78/83 assignments were confirmed (94.0% concordance). Two compounds were misclassified and three remained unassigned, as detailed in Table A7. For Rhubarb, 75 of 83 assignments (90.0%) were confirmed, with two misclassifications and six unassigned compounds (Table A10). For Senna, 47 of 51 assignments (90.3%) were validated, with three misclassified and two unassigned entries (Table A13).

Per-species classification tables (Appendix A Table A3, Table A6, Table A9 and Table A12) provide the full pathway/superclass/class labels and the associated model confidences (Acc. %).

Overall, automated labels closely matched expert curation, indicating that the approach is suitable for high-throughput profiling while retaining chemical interpretability.

A Venn diagram was generated to visualize the overlap between the four species. Only four metabolites—Phloretin (C_15_H_14_O_5_), Kaempferol (C_15_H_10_O_6_), Hispidulin (C_16_H_12_O_6_), and 4-Heptyloxyphenol (C_13_H_20_O_2_)—were common to all extracts, indicating strong chemical specificity for each botanical source. Cascara and Frangula shared 21 metabolites, ten of which were exclusive to these two Rhamnaceae members, whereas Senna and Rhubarb shared 13 compounds, with five uniquely common to both.

**Figure 1:**
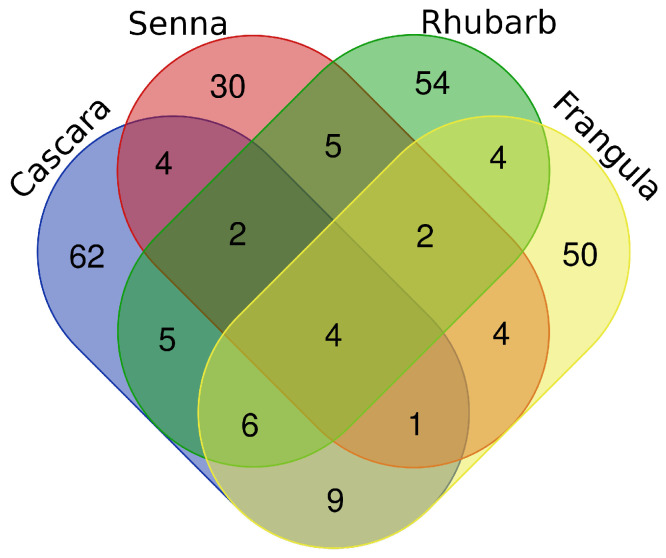
Four-set Venn diagram showing the overlap of annotated metabolite classes among Cascara, Frangula, Rhubarb, and Senna. Each ellipse represents the set of metabolite classes detected in that plant. A class was considered present when at least one metabolite belonging to that class was annotated by LC–MS/MS (MSI Level ≥ 2) in the corresponding sample. Overlapping regions indicate classes shared by two, three, or all four plants, whereas non-overlapping segments represent plant-specific classes. The diagram highlights both the small core of metabolite classes common to all four species and the substantial proportion of classes unique to each plant.

The Venn diagram (Figure 1) further indicated that Cascara and Frangula, belonging to the same family, were similar to each other and showed 21 compounds in common with ten of them characteristic only of these two species. On the other hand, Senna and Rhubarb, seemed to have some metabolites in common, specifically 13 compounds, of which five were exclusive of these two species.

Below, we report a list of the major metabolite classes identified in each plant along with the most abundant metabolites in each category. Moreover, two pie charts were generated for each plant. The first chart summarizes the distribution of identified metabolite superclasses, highlighting the overall chemical profile of the species. The second chart provides a more detailed view of the most abundant category.

### 3.1. Distinctive Metabolites in Cascara

- Polycyclic Aromatic Polyketides: This superclass is represented exclusively by anthraquinones and anthrones, including Emodin (C_15_H_10_O_5_), Aloin A (C_21_H_22_O_9_), Aloin B (C_21_H_22_O_9_), and Cascaroside A (C_27_H_32_O_14_).
- Flavonoids: Abundant representatives include Nobiletin (C_21_H_22_O_8_), Isoliquiritigenin (C_15_H_12_O_4_), Primuletin (C_15_H_10_O_3_), Myrciacitrin V (C_30_H_30_O_13_), Naringin (C_27_H_32_O_14_), and Cirsimarin (C_23_H_24_O_11_).
- Isoflavonoids: Detected examples include Genistein (C_15_H_10_O_5_) and Formononetin (C_16_H_12_O_4_).
- Phenylpropanoids (C6–C3): Represented by Chlorogenic acid (C_16_H_18_O_9_) and Caffeic acid (C_9_H_8_O_4_).
- Tyrosine Alkaloids: Exemplified by Pavine (C_20_H_23_NO_4_).
- Benzenoids: Including 4-Ethylcatechol (C_8_H_10_O_2_).
- Monoterpenoids: Including Demethyloleuropein (C_24_H_30_O_13_).
- Phloroglucinols: Represented by 2,4,6-Trimethoxybenzophenone (C_16_H_16_O_4_).
- Aromatic Polyketides: Including 4-Heptyloxyphenol (C_13_H_20_O_2_).
- Trace compounds: Additional minor representatives of coumarins, diterpenoids, and lignans were also detected.

**Figure 2:**
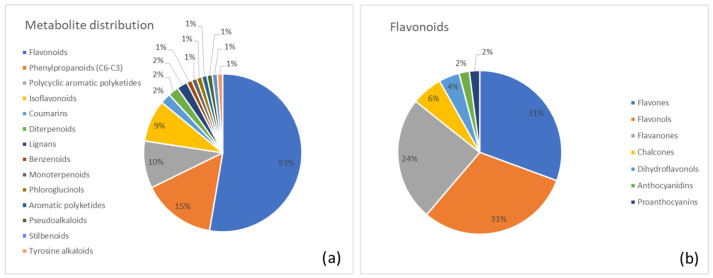
(**a**) Distribution of metabolite classes identified in Cascara. Each slice represents the percentage of annotated metabolites (MSI level ≥ 2) assigned to a given class, according to the structural classification pipeline described in the Methods (GIN-based hierarchical classification). (**b**) Detailed subdivision of the flavonoid superclass in Cascara into the corresponding annotated subclasses. Values are expressed as the percentage of flavonoid metabolites belonging to each subclass.

A clear visualization of the metabolite distribution is shown in Figure 2. In Figure 2a, the pie chart reveals the percentage distribution of major metabolic superclasses in Cascara. The flavonoid class (53%) was clearly the largest, followed by the phenylpropanoids (15%) and anthraquinones (10%). Figure 2b provides a detailed breakdown of the predominant flavonoid class, specifying the contributions of flavones (31%), flavonols (31%), and flavanones (24%).

### 3.2. Distinctive Metabolites in Frangula

- Polycyclic Aromatic Polyketides: Mainly represented by anthraquinones/anthrones, including Emodin (C_15_H_10_O_5_), Glucofrangulin A (C_27_H_30_O_14_), Glucofrangulin B (C_26_H_28_O_14_), Frangulin A (C_21_H_20_O_9_), and Frangulin B (C_20_H_18_O_9_).
- Flavonoids: Liquiritin (C_21_H_22_O_9_), Isoliquiritigenin (C_15_H_12_O_4_), Kaempferol (C_15_H_10_O_6_).
- Isoflavonoids: Genistein (C_15_H_10_O_5_) and Daidzein (C_15_H_10_O_4_).
- Phenylpropanoids (C6–C3): Caffeic acid (C_9_H_8_O_4_), 3-Caffeoylquinic acid (C_16_H_18_O_9_), and Methyl chlorogenate (C_17_H_20_O_9_).
- Monoterpenoids: Oleuropein (C_25_H_32_O_13_), Demethyloleuropein (C_24_H_30_O_13_), and Loganin (C_17_H_26_O_10_) in trace amounts.
- Stilbenoids: Piceatannol (C_14_H_12_O_4_) and Piceid (C_20_H_22_O_8_) in trace amounts.
- Coumarins: 5,6-O-β-D-diglucopyranosylangelicin (C_23_H_26_O_15_).
- Aromatic Polyketides: 4-Heptyloxyphenol (C_13_H_20_O_2_).
- Naphthalenes: Nepodin (C_13_H_12_O_3_).
- Fatty Acids and Conjugates: Oleic acid (C_18_H_34_O_2_) detected in trace amounts (observed only in Frangula).
- Unclassified: Four highly prevalent features grouped as “Other” remained unclassified; accurate *m*/*z* values were observed but spectral/database evidence was insufficient to assign definitive molecular formulas.

**Figure 3:**
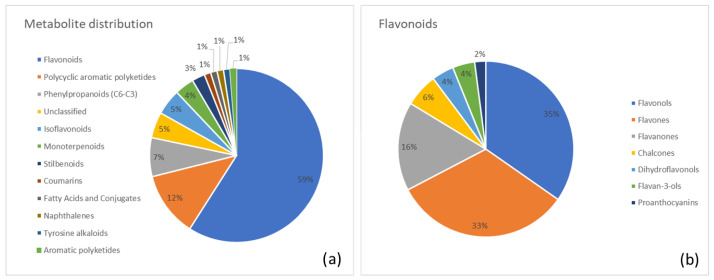
(**a**) Distribution of metabolite classes identified in Frangula. Each slice represents the percentage of annotated metabolites (MSI level ≥ 2) assigned to a given class, according to the structural classification pipeline described in the Methods (GIN-based hierarchical classification). (**b**) Detailed subdivision of the flavonoid superclass in Frangula into the corresponding annotated subclasses. Values are expressed as the percentage of flavonoid metabolites belonging to each subclass.

A distinct visualization of metabolite distribution across superclasses is shown in Figure 3. Figure 3a illustrates the percentage distribution in Frangula, highlighting flavonoids (59%) as the most abundant, followed by polycyclic aromatic polyketides (anthraquinones; 12%) and phenylpropanoids (7%). Figure 3b presents a class-level breakdown of the predominant flavonoid group, with contributions from flavonols (35%), flavones (33%), and flavanones (16%).

### 3.3. Distinctive Metabolites in Rhubarb

#### Superclass-Level Overview

- Polycyclic Aromatic Polyketides: Mainly represented by anthraquinones such as Emodin (C_15_H_10_O_5_), Rhein (C_15_H_8_O_6_), 1,4-Dihydroxyanthraquinone (C_14_H_8_O_4_), and Rhein-8-glucoside (C_21_H_18_O_11_).
- Flavonoids: Abundant representatives include Catechin (C_15_H_14_O_6_).
- Isoflavonoids: Genistein (C_15_H_10_O_5_) and Daidzein (C_15_H_10_O_4_).
- Stilbenoids: Examples are Piceatannol (C_14_H_12_O_4_), Resveratrol (C_14_H_12_O_3_), and Resveratrol 3-O-glucoside (C_20_H_22_O_8_).
- Phenolic Acids: Protocatechuic aldehyde (C_7_H_6_O_3_), Gallic acid (C_7_H_6_O_5_), and Ellagic acid (C_14_H_6_O_8_).
- Chromanes: Including 5-Acetonyl-7-hydroxy-2-methylchromone (C_13_H_12_O_4_) and Aloesin (C_19_H_22_O_9_).
- Naphthalenes: Exemplified by Torachrysone 8-O-β-D-glucoside (C_20_H_24_O_9_).
- Aromatic Polyketides: Including 4-Heptyloxyphenol (C_13_H_20_O_2_).
- Trace Compounds: Minor representatives of diterpenoids and additional phenylpropanoids were also detected.

**Figure 4:**
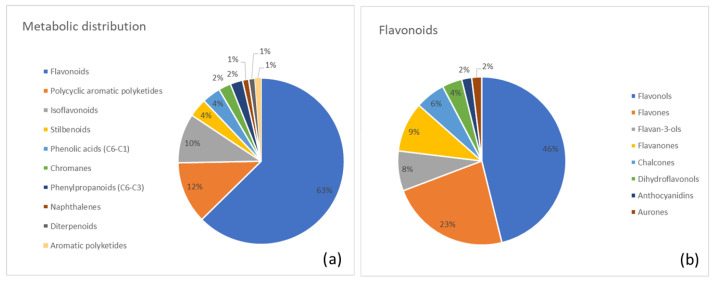
(**a**) Distribution of metabolite classes identified in Rhubarb. Each slice represents the percentage of annotated metabolites (MSI level ≥ 2) assigned to a given class, according to the structural classification pipeline described in the Methods (GIN-based hierarchical classification). (**b**) Detailed subdivision of the flavonoid superclass in Rhubarb into the corresponding annotated subclasses. Values are expressed as the percentage of flavonoid metabolites belonging to each subclass.

The metabolite composition of Rhubarb is summarized in Figure 4. Figure 4a shows the percentage distribution of major metabolite superclasses, highlighting flavonoids (63%) as the predominant group, followed by polycyclic aromatic polyketides (anthraquinones; 12%) and minor contributions from other superclasses. Figure 4b provides a class-level breakdown of the flavonoid superclass, with flavonols (46%), flavones (23%), and flavan-3-ols (8%) representing the most abundant subclasses.

### 3.4. Distinctive Metabolites in Senna

#### Superclass-Level Overview

- Flavonoids: Abundant representatives include Vicenin 2 (C_27_H_30_O_15_), 2′,2 Bisepigallocatechin digallate (C_44_H_34_O_22_), and Luteolin (C_15_H_10_O_6_).
- Isoflavonoids: Demethylwedelolactone (C_15_H_8_O_7_) and Irilone (C_16_H_6_O_3_).
- Polycyclic Aromatic Polyketides: Represented by anthraquinones distinctive of Senna, including Rhein (C_15_H_8_O_6_), Rhein-8-glucoside (C_21_H_18_O_11_), Sennoside A (C_42_H_38_O_20_), and Sennoside B (C_42_H_38_O_20_).
- Phenylpropanoids (C6–C3): Examples include Guaethol (C_8_H_10_O_2_) and Eugenol (C_10_H_12_O_2_).
- Coumarins: Exemplified by 11-O-Galloylbergenin (C_21_H_20_O_13_).
- Benzenoids: Including Creosol (C_8_H_10_O_2_).
- Aromatic Polyketides: Including 4-Heptyloxyphenol (C_13_H_20_O_2_), also observed in the other species.
- Trace Compounds: Minor representatives of diterpenoids, lignans, and naphthalenes were also detected.

**Figure 5:**
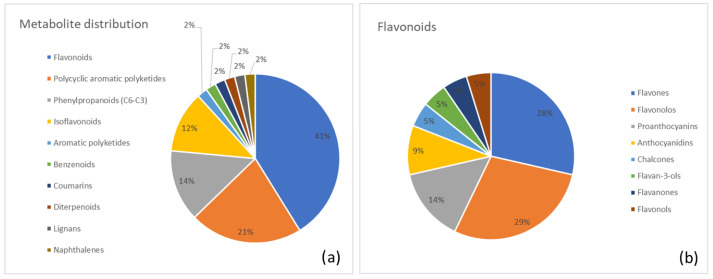
(**a**) Distribution of metabolite classes identified in Senna. Each slice represents the percentage of annotated metabolites (MSI level ≥ 2) assigned to a given class, according to the structural classification pipeline described in the Methods (GIN-based hierarchical classification). (**b**) Detailed subdivision of the flavonoid superclass in Senna into the corresponding annotated subclasses. Values are expressed as the percentage of flavonoid metabolites belonging to each subclass.

A clear representation of the metabolite distribution in Senna is provided in Figure 5. Figure 5a shows the percentage distribution of major metabolite superclasses, with flavonoids (41%) as the most abundant group, followed by polycyclic aromatic polyketides (anthraquinones; 21%) and phenylpropanoid (12%). Figure 5b presents a class-level breakdown of the flavonoid superclass, highlighting the relative contributions of flavones (29%), flavonols (28%), and proanthocyanins (14%).

Taken together, the hierarchical classification and species-specific metabolite distributions provide a comprehensive overview of the chemical diversity across the four plants. The distribution of major compound superclasses is summarized in Table 1.

**Figure 6:**
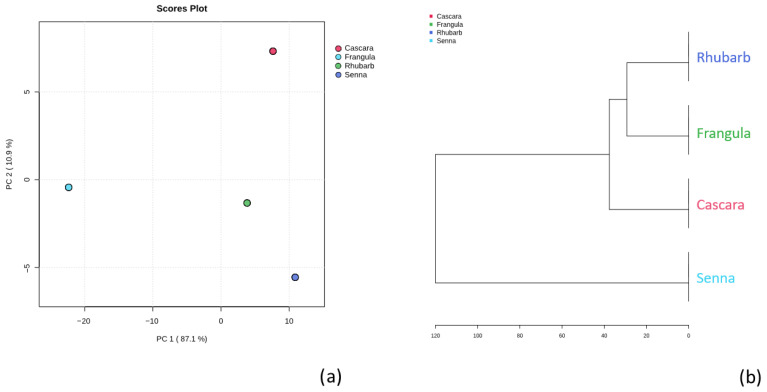
(**a**) Principal component analysis (PCA) score plot of the metabolomic profiles of Cascara, Frangula, Rhubarb, and Senna. Data were autoscaled (z-score normalization). PC1 explains 87.1% of the total variance and PC2 explains 10.9%. Each point represents a technical replicate, and samples are colour-coded by species (Cascara: red; Frangula: green; Senna: light blue; Rhubarb: blue). Cascara clusters to the right along PC1 and Senna to the left, while Frangula and Rhubarb occupy the lower-right quadrant, reflecting interspecies differences in overall metabolite composition. (**b**) Hierarchical cluster analysis (HCA) dendrogram of the same samples based on Euclidean distance. Colours correspond to the PCA groups. Rhubarb and Frangula cluster first and then merge with Cascara, while Senna forms the most distant branch. The horizontal axis represents dissimilarity (0–120).

Principal component analysis (PCA) was applied to the normalized metabolite dataset in order to explore the interspecies variability. The first two principal components accounted for 87.1% (PC1) and 10.9% (PC2) of total variance, respectively. The PCA score plot (Figure 6a) reveals a clear separation of Senna from the other species, indicating a distinct metabolomic signature.

Hierarchical cluster analysis (HCA) based on the Euclidean distance (Figure 6b) produced comparable results: Frangula and Rhubarb clustered closely, followed by Cascara, whereas Senna formed an independent branch, confirming its unique chemical composition.

**Figure 7:**
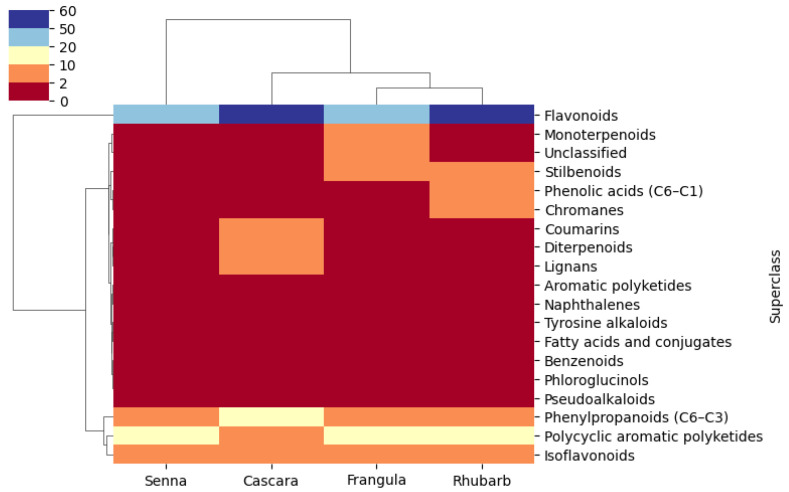
Heatmap showing the distribution of 19 metabolite superclasses across the analysed samples. Each row corresponds to a distinct superclass and each column to a single LC–MS/MS analysis (technical replicate), grouped by plant species. The colour scale ranges from red (low relative abundance, value = 0) to blue (high relative abundance, maximum value = 60; not further normalized). Unsupervised hierarchical clustering was applied to both rows and columns using the Euclidean distance and average linkage; the corresponding dendrograms are displayed alongside the heatmap. The plot highlights a clear predominance of flavonoids, which are the only superclass showing consistently high abundance across all samples, whereas the remaining superclasses display moderate to low levels, reflected by intermediate shades between red and blue.

A heatmap representation (Figure 7) illustrates the distribution of metabolites across classes. Two main clusters can be observed: the first is dominated by flavonoids common to all plants, while the second is subdivided into two subclusters: (i) phenylpropanoids, anthraquinones, and anthrones, suggesting shared biosynthetic pathways; and (ii) additional metabolic groups such as aromatic polyketides, chromanes, stilbenoids, and coumarins, reflecting high chemical diversity and species-specific biosynthetic specialization.

This compositional diversity contributes to the distinct biochemical identity observed for each plant species.

## 4. Discussion

A chemical fingerprint profile can comprehensively reflect the types of chemical components contained in medicinal plants and their products, which can then be used to describe and evaluate their quality as a whole [34]. To this end, a method was developed by combining the high separation performance of HPLC applied to complex samples with the high selectivity and sensitivity of MS, allowing for a comprehensive evaluation of the medicinal plants under investigation. In fact, by contributing to a better understanding of the distribution and variability of these compounds within species, plant metabolomics provides a formidable resource for exploring the richness and complexity of metabolites found in plants.

The GIN-based hierarchical classification proved robust across the dataset. Close inspection of the few discordant cases revealed chemically plausible failure modes that are typical in large-scale automated annotation. These included ambiguities between structurally similar scaffolds, misclassification driven by partial structural features (e.g., side-chain length, oxidation state, or ring substitution patterns), and limited representation of certain compound families in the training data. Such discrepancies were readily resolved by expert review and did not alter the overall conclusions at the pathway or superclass level.

Confidence values were highly informative: assignments above 99% were almost invariably confirmed, while discordant cases showed lower or imbalanced scores across hierarchy levels. This supports a pragmatic workflow in which automated predictions are retained as defaults and selectively curated when confidence flags emerge. Such an approach balances throughput with accuracy, preserves reproducibility, and minimizes the risk of propagating annotation errors. Studies integrating untargeted metabolomics with transparent uncertainty estimates and expert validation remain relatively uncommon, underscoring current limitations in evaluating the efficacy and safety of plant-derived products [35].

Using this framework, we identified 93 compounds in Cascara, 83 in Rhubarb, 83 in Frangula, and 51 in Senna.

Among the identified metabolites (MSI level 2 unless otherwise specified), several species-enriched features emerged in our dataset. In particular, Pavine, a tyrosine alkaloid, was detected in Cascara and Frangula preparations; to the best of our knowledge, this represents its first report in these herbal drugs. Likewise, Vicenin 2 was observed in Senna, whereas Piceatannol showed comparatively higher abundances in Rhubarb. These assignments are based on high-resolution MS and MS/MS database matching, and as such should be regarded as putative annotations pending confirmation with authentic standards. In all four plants, flavonoids represented the dominant superclass, confirming their central role in the phytochemical composition of these species, which is in agreement with previous studies [36,37,38,39].

With regard to HADs, each plant was characterized by compounds typical of the species itself, confirming what has previously been stated in several studies [18,19,20,23]. Despite the complexity of the analysed samples, their natural variability, and their origin from different plant parts (e.g., leaves and bark), it was possible to confirm consistent, species-specific trends in HAD composition [15]. In Frangula, the trend was defined by a predominance of frangulins and glucofrangulins A and B, followed by emodin. In Cascara, the profile was marked by cascaroside A, aloin A, and aloin B, with emodin and traces of aloe-emodin. In Rhubarb, rhein and rhein-8-glucoside dominated together with emodin, while Senna was characterized by abundant sennidins (A and B), sennosides (A and B), and rhein derivatives. These profiles illustrate not only the expected complexity of HAD distribution but also the taxonomic consistency across species despite differences in the analysed plant parts.

From a pharmacological perspective, the observed chemical diversity is expected to influence both bioavailability and safety. For instance, glycosylated hydroxyanthracene derivatives generally require metabolic activation in the gut before absorption, whereas aglycones and low-molecular-weight phenolics are typically more readily absorbed but may also display different toxicity profiles [13]. Likewise, flavonoids and other polyphenols can modulate intestinal permeability, metabolism, and oxidative stress, potentially affecting the overall response to these preparations [40]. However, the present study was not designed to directly evaluate bioavailability or safety, and no pharmacokinetic or toxicological measurements were performed. Therefore, our data should be interpreted as a comprehensive chemical framework that can inform future functional studies, rather than as a direct assessment of clinical efficacy or risk.

The data highlight the significant differences in the composition of compound classes among the plants. Cascara and Frangula, both of which belong to the Rhamnaceae family, exhibit notable similarities, especially in the abundance of flavonoids and anthraquinones. Rhubarb, on the other hand, is characterized by a higher presence of phenolic acids and chromanes. Senna stands out with a distinct distribution, particularly in terms of coumarins and specific monoterpenoids. This comparative view emphasizes the metabolic diversity among the species and highlights both family-specific and species-specific chemical signatures.

An additional key observation is the limited number of metabolites shared among the four species. The Venn diagram analysis vividly illustrates that only four metabolites are common across all plants. This scarcity of shared compounds indicates a high degree of specificity in the secondary metabolites present in each species. This observation prompted us to further explore inter-species relationships through multivariate approaches.

Our statistical analysis, including principal component analysis (PCA) and hierarchical cluster analysis (HCA), provided a deeper understanding of the metabolic relationships among the plant samples. Senna emerged as distinct, reinforcing its unique metabolic profile compared to the other varieties. Moreover, the heatmap analysis highlighted two main clusters, revealing the prevalence of flavonoids across all plants and indicating potential biosynthetic connections in the pathways of phenylpropanoids and anthraquinones. The complex interrelationships among the various metabolites that make up the bigger subcluster highlight the intricacy and interconnectivity of these plants’ metabolic profiles.

## 5. Conclusions

This study provides a comprehensive metabolomic characterization of four medicinal plants widely used for their laxative properties: Cascara (*Rhamnus purshiana*), Senna (*Cassia angustifolia*), Rhubarb (*Rheum palmatum*), and Frangula (*Rhamnus frangula*). By combining untargeted LC–MS/MS with bioinformatic approaches, we generated a high-resolution chemical fingerprint that extends beyond the well-known hydroxyanthracene derivatives (HADs).

The analysis revealed species-specific metabolic signatures, including the first report of Pavine in the Rhamnaceae family, and highlighted the predominance of flavonoids, anthraquinones, phenylpropanoids, and other bioactive classes. Strikingly, only four flavonoids were shared across all species, underscoring the remarkable biochemical diversity and taxonomic specificity of these plants. Our approach proved effective for fingerprinting complex botanical matrices and offers a robust framework for the discovery of distinctive metabolites with potential biological relevance. Accurate structural classification not only deepens our understanding of plant metabolic diversity but also provides a chemical basis for evaluating the safety and efficacy of phytotherapeutic preparations. Future studies combining metabolomic profiling with bioactivity assays and biosynthetic pathway analysis will be crucial in translating these findings into pharmacological applications in order to assess the therapeutic potential of these plants.

## Figures and Tables

**Table 1:** Distribution of major compound superclasses across Cascara, Frangula, Rhubarb, and Senna.

Class	Cascara	Frangula	Rhubarb	Senna
Flavonoids	50	49	52	21
Phenylpropanoids (C6–C3)	14	6	2	7
Polycyclic aromatic polyketides	9	10	10	11
Isoflavonoids	7	4	8	6
Coumarins	2	1	–	1
Diterpenoids	2	–	1	1
Lignans	2	–	–	1
Benzenoids	1	–	–	1
Monoterpenoids	1	3	–	–
Phloroglucinols	1	–	–	–
Aromatic polyketides	1	1	1	1
Pseudoalkaloids	1	–	–	–
Stilbenoids	1	2	3	–
Tyrosine alkaloids	1	1	–	–
Fatty acids and conjugates	–	1	–	–
Naphthalenes	–	1	1	1
Phenolic acids (C6–C1)	–	–	3	–
Chromanes	–	–	2	–
Unclassified	–	4	–	–

## Data Availability

All processed metabolomic data supporting this study are provided in the Supplementary Materials as Excel tables exported from Compound Discoverer, including for each detected feature the precursor *m*/*z*, retention time, peak area, ionization mode, database-derived annotation, and MSI confidence level. The full implementation of the graph-based neural network used for metabolite classification (model architectures, trained weights and analysis scripts) is openly available in the associated GitHub repository: https://github.com/bcorrad/ginestra25 (accessed on 20 November 2025).

## Supplementary Materials

The following supporting information can be downloaded at https://www.mdpi.com/article/10.3390/metabo15120779/s1, Table S1: Excel file reporting, for each detected feature in Cascara, the following fields: Correspondent name, molecular formula, annotation mass error (DeltaMass, ppm), molecular weight, *m*/*z*, retention time (min), maximum peak area, MSI level, MS2 information, and reference ion; Table S2: Excel file reporting, for each detected feature in Frangula, the same set of fields; Table S3: Excel file reporting, for each detected feature in Rhubarb, the same set of fields; Table S4: Excel file reporting, for each detected feature in Senna, the same set of fields.

## Abbreviations

The following abbreviations are used in this manuscript:

HADs	Hydroxyanthracene Derivatives
UPLC–MS/MS	Ultra-High-Performance Liquid Chromatography–Tandem Mass Spectrometry
HCA	Hierarchical Cluster Analysis
GIN	Graph Isomorphism Network

## Appendix A

Appendix A reports the model architecture and the complete set of tables for each species.

### Appendix A.1. Model Architecture

The GIN (Graph Isomorphism Network) architectures reported in Table A1 were used for hierarchical metabolite classification at the pathway, superclass, and class levels. Each model processes molecular graphs using successive GIN convolutional layers followed by batch normalization and fully connected layers. Output dimensions correspond to the number of categories at each hierarchy level (7, 70, and 653, respectively).

**Table A1:** Layer-by-layer GIN architectures for pathway, superclass, and class classification tasks.

Model	Pathway		Superclass		Class	
	**Layer**	**Params**	**Layer**	**Params**	**Layer**	**Params**
	GINConv	(23, 512)	GINConv	(23, 512)	GINConv	(23, 512)
	BatchNorm1d	(512)	BatchNorm1d	(512)	BatchNorm1d	(512)
	GINConv ×3	(512, 512)	GINConv ×3	(512, 512)	GINConv ×2	(512, 512)
	BatchNorm1d	(512)	BatchNorm1d	(512)	BatchNorm1d	(512)
GIN	Linear	(2560, 1024)	Linear	(2560, 1024)	Linear	(2048, 1024)
	BatchNorm1d	(1024)	BatchNorm1d	(1024)	BatchNorm1d	(1024)
	Output layer	(1024, 7)	Output layer	(1024, 70)	Output layer	(1024, 653)

### Appendix A.2. Cascara

For Cascara, Table A2 lists all identified metabolites with validated superclass assignments, while Table A3 provides the hierarchical classification with model-derived confidence scores. Table A4 highlights the few discordant cases, which are discussed further below. For full-precision *m*/*z* values (reported to four decimal places) together with the corresponding retention times (RT) and mass errors (Δ, ppm), readers are referred to Supplementary Table S1.

**Table A2:** Cascara—Identified metabolites with validated *Superclass*. Molecular weights are rounded to two decimals.

Component	Formula	Molecular Weight	Superclass
Pavine	C_20_H_23_NO_4_	341.16	Tyrosine alkaloids
Emodin	C_15_H_10_O_5_	270.05	Polycyclic aromatic polyketides
Aloin A	C_21_H_22_O_9_	418.13	Polycyclic aromatic polyketides
Nobiletin	C_21_H_22_O_8_	402.13	Flavonoids
Aloin B	C_21_H_22_O_9_	418.13	Polycyclic aromatic polyketides
Isoliquiritigenin	C_15_H_12_O_4_	256.07	Flavonoids
2-Ethyl-9,10-anthraquinone	C_16_H_12_O_2_	236.08	Polycyclic aromatic polyketides
Primuletin	C_15_H_10_O_3_	238.06	Flavonoids
Cascaroside A	C_27_H_32_O_14_	580.18	Polycyclic aromatic polyketides
myrciacitrin V	C_30_H_30_O_13_	598.17	Flavonoids
Naringin	C_27_H_32_O_14_	580.18	Flavonoids
Cascaroside C/Cascaroside D	C_27_H_32_O_13_	564.19	Polycyclic aromatic polyketides
Cirsimarin	C_23_H_24_O_11_	476.13	Flavonoids
myrciacitrin IV	C_32_H_32_O_13_	624.19	Flavonoids
Demethyloleuropein	C_24_H_30_O_13_	526.17	Monoterpenoids
Flavone	C_15_H_10_O_2_	222.07	Flavonoids
1,8-Dihydroxy-9(10H)-anthracenone	C_14_H_10_O_3_	226.06	Polycyclic aromatic polyketides
Kaempferol	C_15_H_10_O_6_	286.05	Flavonoids
Chrysoeriol 7-O-glucoside	C_22_H_22_O_11_	462.12	Flavonoids
Narirutin $4^{\prime }$-O-glucoside	C_33_H_42_O_19_	742.23	Flavonoids
Narirutin	C_27_H_32_O_14_	580.18	Flavonoids
Chlorogenic acid	C_16_H_18_O_9_	354.09	Phenylpropanoids (C6–C3)
Caffeic acid	C_9_H_8_O_4_	180.04	Phenylpropanoids (C6–C3)
Aloe emodin	C_15_H_10_O_5_	254.06	Polycyclic aromatic polyketides
3-Feruloylquinic acid	C_17_H_20_O_9_	368.11	Phenylpropanoids (C6–C3)
Genistein	C_15_H_10_O_5_	270.05	Isoflavonoids
Rhoifolin $4^{\prime }$-O-glucoside	C_33_H_40_O_19_	740.22	Flavonoids
Procyanidin dimer B1	C_30_H_26_O_12_	578.14	Flavonoids
Formononetin	C_16_H_12_O_4_	268.07	Isoflavonoids
Luteolin	C_15_H_10_O_6_	286.05	Flavonoids
Prunin	C_21_H_22_O_10_	434.12	Flavonoids
Kaempferol 3-O-glucosyl-rhamnosyl-galactoside	C_33_H_40_O_20_	756.21	Flavonoids
Nepetin	C_16_H_12_O_7_	316.06	Flavonoids
3-Caffeoylquinic acid	C_16_H_18_O_9_	354.10	Phenylpropanoids (C6–C3)
$6^{\prime \prime }$-O-Acetylglycitin	C_24_H_24_O_11_	488.13	Isoflavonoids
2,4,6-Trimethoxybenzophenone	C_16_H_16_O_4_	272.11	Phloroglucinols
Daidzin	C_21_H_20_O_9_	416.11	Isoflavonoids
Geraldone	C_16_H_12_O_5_	284.07	Flavonoids
6-Methoxyflavonol	C_16_H_12_O_4_	268.07	Flavonoids
1-Sinapoyl-2-feruloylgentiobiose	C_33_H_40_O_18_	724.22	Phenylpropanoids (C6–C3)
3-Methoxynobiletin	C_22_H_24_O_9_	432.14	Flavonoids
1,2-Diferuloylgentiobiose	C_32_H_38_O_17_	694.21	Phenylpropanoids (C6–C3)
Quercetin	C_15_H_10_O_7_	302.04	Flavonoids
Phloretin	C_15_H_14_O_5_	274.08	Flavonoids
Galangin	C_15_H_10_O_5_	270.05	Flavonoids
Coumestrol	C_15_H_8_O_5_	268.04	Isoflavonoids
Apocynin	C_9_H_10_O_3_	166.06	Phenylpropanoids (C6–C3)
Phloridzin	C_21_H_24_O_10_	436.14	Flavonoids
Sinensetin	C_20_H_20_O_7_	372.12	Flavonoids
Eriodictyol	C_15_H_12_O_6_	288.06	Flavonoids
Tectoridin	C_22_H_22_O_11_	462.12	Isoflavonoids
Chrysin	C_15_H_10_O_4_	254.06	Flavonoids
Taxifolin	C_15_H_12_O_7_	304.06	Flavonoids
Vanillin	C_8_H_8_O_3_	152.05	Phenylpropanoids (C6–C3)
Cyanidin 3-O-glucosyl-rutinoside	C_33_H_41_O_20_	757.22	Flavonoids
Sinapaldehyde	C_11_H_12_O_4_	208.07	Phenylpropanoids (C6–C3)
3-hydroxyflavanone	C_15_H_12_O_3_	240.08	Flavonoids
Eugenol	C_10_H_12_O_2_	164.08	Phenylpropanoids (C6–C3)
p-Coumaric acid ethyl ester	C_11_H_12_O_3_	192.08	Phenylpropanoids (C6–C3)
Carnosol	C_20_H_26_O_4_	330.18	Diterpenoids
isoquercetin	C_21_H_20_O_12_	464.10	Flavonoids
3-p-Coumaroylquinic acid	C_16_H_18_O_8_	338.10	Phenylpropanoids (C6–C3)
Morin	C_15_H_10_O_7_	302.04	Flavonoids
p-Coumaroyl glycolic acid	C_11_H_10_O_5_	222.05	Phenylpropanoids (C6–C3)
Homoeriodictyol	C_16_H_14_O_6_	302.08	Flavonoids
4-Vinylguaiacol	C_9_H_10_O_2_	150.07	Phenylpropanoids (C6–C3)
Apigenin 7-O-($6^{\prime \prime }$-malonyl-apiosyl-glucoside)	C_29_H_30_O_17_	650.15	Flavonoids
Kaempferitrin	C_27_H_30_O_14_	578.16	Flavonoids
olmelin	C_16_H_12_O_5_	284.07	Isoflavonoids
Coumarin	C_9_H_6_O_2_	146.04	Coumarins
Hesperetin	C_16_H_14_O_6_	302.08	Flavonoids
m-Coumaric acid	C_9_H_8_O_3_	164.05	Phenylpropanoids (C6–C3)
3,4,7,8-TETRAHYDROXYFLAVONE	C_15_H_10_O_6_	286.05	Monoterpenoids
$6^{\prime \prime }$-O-Malonyldaidzin	C_24_H_22_O_12_	502.11	Isoflavonoids
Hispidulin	C_16_H_12_O_6_	300.06	Flavonoids
Apigenin	C_15_H_10_O_5_	270.05	Flavonoids
Syringaresinol	C_22_H_26_O_8_	418.16	Lignans
Secoisolariciresinol	C_20_H_26_O_6_	362.17	Lignans
Kalambroside B	C_30_H_34_O_17_	666.18	Flavonoids
Naringenin 7-O-glucoside	C_21_H_22_O_10_	434.12	Flavonoids
Scopoletin	C_10_H_8_O_4_	192.04	Coumarins
Kaempferol 3-O-β-rutinoside	C_27_H_30_O_15_	594.16	Flavonoids
Acetyl eugenol	C_12_H_14_O_3_	206.09	Phenylpropanoids (C6–C3)
Didymin/Poncirin	C_28_H_34_O_14_	594.20	Flavonoids
1,2-Disinapoylgentiobiose	C_34_H_42_O_19_	754.23	Phenylpropanoids (C6–C3)
Carnosic acid	C_20_H_28_O_4_	332.20	Diterpenoids
Dihydroquercetin 3-O-rhamnoside	C_21_H_22_O_11_	450.12	Flavonoids
Isorhamnetin 3-O-glucoside 7-O-rhamnoside	C_28_H_32_O_16_	624.17	Flavonoids
Quercetin 3-O-xylosyl-rutinoside	C_32_H_38_O_20_	742.20	Flavonoids
2-O-Rhamnosylvitexin	C_27_H_30_O_14_	578.16	Flavonoids
4-Ethylcatechol	C_8_H_10_O_2_	138.07	Benzenoids
9,10-Dihydroxyanthracene	C_14_H_10_O_2_	210.07	Polycyclic aromatic polyketides
4-Heptyloxyphenol	C_13_H_20_O_2_	208.15	Aromatic polyketides

**Table A3:** Cascara—Hierarchical classification of identified metabolites with model accuracy expressed as percentage (Acc. %).

Component	Pathway	Acc.%	Superclass	Acc.%	Class	Acc.%
Pavine	Alkaloids	99.99	Tyrosine alkaloids	100.00	Isoquinoline alkaloids	88.90
Emodin	Polyketides	99.90	Polycyclic aromatic polyketides	100.00	Anthraquinones and anthrones	99.90
Aloin A	Polyketides	99.80	Polycyclic aromatic polyketides	99.90	Anthraquinones and anthrones	99.90
Nobiletin	Shikimates and Phenylpropanoids	99.97	Flavonoids	100.00	Flavones	99.98
Aloin B	Polyketides	99.80	Polycyclic aromatic polyketides	99.90	Anthraquinones and anthrones	99.90
Isoliquiritigenin	Shikimates and Phenylpropanoids	99.99	Flavonoids	100.00	Chalcones	100.00
2-Ethyl-9,10-anthraquinone	Polyketides	100.00	Polycyclic aromatic polyketides	100.00	Anthraquinones and anthrones	100.00
Primuletin	Shikimates and Phenylpropanoids	98.60	Flavonoids	100.00	Flavones	100.00
Cascaroside A	Polyketides	99.70	Polycyclic aromatic polyketides	99.90	Anthraquinones and anthrones	99.70
myrciacitrin V	Shikimates and Phenylpropanoids	98.70	Flavonoids	100.00	Flavanones	100.00
Naringin	Shikimates and Phenylpropanoids	100.00	Flavonoids	100.00	Flavanones	100.00
Cascaroside C/Cascaroside D	Polyketides	99.60	Polycyclic aromatic polyketides	100.00	Anthraquinones and anthrones	99.70
Cirsimarin	Shikimates and Phenylpropanoids	100.00	Flavonoids	100.00	Flavones	100.00
myrciacitrin IV	Shikimates and Phenylpropanoids	99.80	Flavonoids	100.00	Flavanones	100.00
Demethyloleuropein	Terpenoids	100.00	Monoterpenoids	100.00	Secoiridoid monoterpenoids	100.00
Flavone	Shikimates and Phenylpropanoids	99.50	Flavonoids	100.00	Flavones	100.00
1,8-Dihydroxy-9(10H)-anthracenone	Polyketides	100.00	Polycyclic aromatic polyketides	100.00	Anthraquinones and anthrones	100.00
Kaempferol	Shikimates and Phenylpropanoids	100.00	Flavonoids	100.00	Flavonols	100.00
Chrysoeriol 7-O-glucoside	Shikimates and Phenylpropanoids	100.00	Flavonoids	100.00	Flavones	100.00
Narirutin $4^{\prime }$-O-glucoside	Shikimates and Phenylpropanoids	100.00	Flavonoids	100.00	Flavanones	100.00
Narirutin	Shikimates and Phenylpropanoids	100.00	Flavonoids	100.00	Flavanones	100.00
Chlorogenic acid	Shikimates and Phenylpropanoids	99.80	Phenylpropanoids (C6–C3)	97.10	Cinnamic acids and derivatives	99.50
Caffeic acid	Shikimates and Phenylpropanoids	98.70	Phenylpropanoids (C6–C3)	96.30	Cinnamic acids and derivatives	98.20
Aloe emodin	Polyketides	100.00	Polycyclic aromatic polyketides	100.00	Anthraquinones and anthrones	100.00
3-Feruloylquinic acid	Shikimates and Phenylpropanoids	99.70	Phenylpropanoids (C6–C3)	98.70	Cinnamic acids and derivatives	99.80
Genistein	Shikimates and Phenylpropanoids	99.80	Isoflavonoids	100.00	Isoflavones	99.90
Rhoifolin $4^{\prime }$-O-glucoside	Shikimates and Phenylpropanoids	100.00	Flavonoids	100.00	Flavones	100.00
Procyanidin dimer B1	Shikimates and Phenylpropanoids	100.00	Flavonoids	100.00	Proanthocyanins	99.90
Formononetin	Shikimates and Phenylpropanoids	99.90	Isoflavonoids	100.00	Isoflavones	99.90
Luteolin	Shikimates and Phenylpropanoids	99.90	Flavonoids	100.00	Flavones	100.00
Prunin	Shikimates and Phenylpropanoids	100.00	Flavonoids	100.00	Flavanones	100.00
Kaempferol 3-O-glucosyl-rhamnosyl-galactoside	Shikimates and Phenylpropanoids	100.00	Flavonoids	100.00	Flavonols	100.00
Nepetin	Shikimates and Phenylpropanoids	100.00	Flavonoids	100.00	Flavones	100.00
3-Caffeoylquinic acid	Shikimates and Phenylpropanoids	99.80	Phenylpropanoids (C6–C3)	97.10	Cinnamic acids and derivatives	99.50
$6^{\prime \prime }$-O-Acetylglycitin	Shikimates and Phenylpropanoids	100.00	Isoflavonoids	100.00	Isoflavones	100.00
2,4,6-Trimethoxybenzophenone	Polyketides	99.60	Phloroglucinols	100.00	Acyl phloroglucinols	99.90
Daidzin	Shikimates and Phenylpropanoids	100.00	Isoflavonoids	100.00	Isoflavones	100.00
Geraldone	Shikimates and Phenylpropanoids	100.00	Flavonoids	100.00	Flavones	100.00
6-Methoxyflavonol	Shikimates and Phenylpropanoids	99.90	Flavonoids	100.00	Flavonols	100.00
1-Sinapoyl-2-feruloylgentiobiose	Shikimates and Phenylpropanoids	94.00	Phenylpropanoids (C6–C3)	99.70	Cinnamic acids and derivatives	99.90
3-Methoxynobiletin	Shikimates and Phenylpropanoids	100.00	Flavonoids	100.00	Flavonols	100.00
1,2-Diferuloylgentiobiose	Shikimates and Phenylpropanoids	92.90	Phenylpropanoids (C6–C3)	99.80	Cinnamic acids and derivatives	100.00
Quercetin	Shikimates and Phenylpropanoids	99.90	Flavonoids	100.00	Flavonols	100.00
Phloretin	Shikimates and Phenylpropanoids	99.10	Flavonoids	100.00	Chalcones	100.00
Galangin	Shikimates and Phenylpropanoids	99.70	Flavonoids	100.00	Flavonols	100.00
Coumestrol	Shikimates and Phenylpropanoids	99.90	Isoflavonoids	99.80	Coumestan	99.80
Apocynin	Shikimates and Phenylpropanoids	98.80	Phenylpropanoids (C6–C3)	56.60	Simple phenolic acids	31.00
Phloridzin	Shikimates and Phenylpropanoids	99.60	Flavonoids	100.00	Chalcones	99.90
Sinensetin	Shikimates and Phenylpropanoids	100.00	Flavonoids	100.00	Flavones	100.00
Eriodictyol	Shikimates and Phenylpropanoids	100.00	Flavonoids	100.00	Flavanones	99.80
Tectoridin	Shikimates and Phenylpropanoids	100.00	Isoflavonoids	100.00	Isoflavones	99.90
Chrysin	Shikimates and Phenylpropanoids	99.50	Flavonoids	100.00	Flavones	100.00
Taxifolin	Shikimates and Phenylpropanoids	100.00	Flavonoids	100.00	Dihydroflavonols	99.80
Cyanidin 3-O-glucosyl-rutinoside	Shikimates and Phenylpropanoids	100.00	Flavonoids	100.00	Anthocyanidins	100.00
Sinapaldehyde	Shikimates and Phenylpropanoids	98.00	Phenylpropanoids (C6–C3)	91.40	Cinnamic acids and derivatives	29.20
3-hydroxyflavanone	Shikimates and Phenylpropanoids	100.00	Flavonoids	100.00	Flavanones	99.90
Eugenol	Shikimates and Phenylpropanoids	100.00	Phenylpropanoids (C6–C3)	98.80	Cinnamic acids and derivatives	96.30
p-Coumaric acid ethyl ester	Shikimates and Phenylpropanoids	98.70	Phenylpropanoids (C6–C3)	97.00	Cinnamic acids and derivatives	95.90
Carnosol	Terpenoids	100.00	Diterpenoids	100.00	Abietane diterpenoids	99.90
isoquercetin	Shikimates and Phenylpropanoids	100.00	Flavonoids	100.00	Flavonols	100.00
3-p-Coumaroylquinic acid	Shikimates and Phenylpropanoids	100.00	Phenylpropanoids (C6–C3)	94.20	Cinnamic acids and derivatives	99.60
Morin	Shikimates and Phenylpropanoids	99.70	Flavonoids	100.00	Flavonols	100.00
p-Coumaroyl glycolic acid	Shikimates and Phenylpropanoids	99.10	Phenylpropanoids (C6–C3)	15.60	Cinnamic acids and derivatives	57.70
Homoeriodictyol	Shikimates and Phenylpropanoids	100.00	Flavonoids	99.90	Flavanones	99.80
4-Vinylguaiacol	Shikimates and Phenylpropanoids	100.00	Phenylpropanoids (C6–C3)	90.00	Cinnamic acids and derivatives	60.60
Apigenin 7-O-($6^{\prime \prime }$-malonyl-apiosyl-glucoside)	Shikimates and Phenylpropanoids	100.00	Flavonoids	100.00	Flavones	99.90
Kaempferitrin	Shikimates and Phenylpropanoids	100.00	Flavonoids	100.00	Flavonols	100.00
olmelin	Shikimates and Phenylpropanoids	99.80	Isoflavonoids	100.00	Isoflavones	99.90
Coumarin	Shikimates and Phenylpropanoids	99.90	Coumarins	99.80	Simple coumarins	90.80
Hesperetin	Shikimates and Phenylpropanoids	100.00	Flavonoids	99.90	Flavanones	99.80
m-Coumaric acid	Shikimates and Phenylpropanoids	99.30	Phenylpropanoids (C6–C3)	91.90	Cinnamic acids and derivatives	99.00
3,4,7,8-TETRAHYDROXYFLAVONE	Terpenoids	48.10	Monoterpenoids	70.20	Simple diketopiperazine alkaloids	7.80
$6^{\prime \prime }$-O-Malonyldaidzin	Shikimates and Phenylpropanoids	100.00	Isoflavonoids	99.90	Isoflavones	99.90
Hispidulin	Shikimates and Phenylpropanoids	100.00	Flavonoids	100.00	Flavones	100.00
Apigenin	Shikimates and Phenylpropanoids	99.90	Flavonoids	100.00	Flavones	100.00
Syringaresinol	Shikimates and Phenylpropanoids	100.00	Lignans	100.00	Furofuranoid lignans	100.00
Secoisolariciresinol	Shikimates and Phenylpropanoids	100.00	Lignans	100.00	Dibenzylbutane lignans	100.00
Kalambroside B	Shikimates and Phenylpropanoids	100.00	Flavonoids	100.00	Flavonols	100.00
Naringenin 7-O-glucoside	Shikimates and Phenylpropanoids	100.00	Flavonoids	100.00	Flavanones	100.00
Scopoletin	Shikimates and Phenylpropanoids	100.00	Coumarins	99.80	Simple coumarins	95.60
Kaempferol 3-O-β-rutinoside	Shikimates and Phenylpropanoids	100.00	Flavonoids	100.00	Flavonols	100.00
Acetyl eugenol	Shikimates and Phenylpropanoids	99.90	Phenylpropanoids (C6–C3)	99.30	Cinnamic acids and derivatives	95.80
Didymin/Poncirin	Shikimates and Phenylpropanoids	100.00	Flavonoids	99.90	Flavanones	99.90
1,2-Disinapoylgentiobiose	Shikimates and Phenylpropanoids	95.40	Phenylpropanoids (C6–C3)	99.50	Cinnamic acids and derivatives	100.00
Carnosic acid	Terpenoids	100.00	Diterpenoids	100.00	Abietane diterpenoids	99.70
Dihydroquercetin 3-O-rhamnoside	Shikimates and Phenylpropanoids	100.00	Flavonoids	99.90	Dihydroflavonols	99.70
Isorhamnetin 3-O-glucoside 7-O-rhamnoside	Shikimates and Phenylpropanoids	100.00	Flavonoids	100.00	Flavonols	100.00
Quercetin 3-O-xylosyl-rutinoside	Shikimates and Phenylpropanoids	100.00	Flavonoids	100.00	Flavonols	100.00
2-O-Rhamnosylvitexin	Shikimates and Phenylpropanoids	100.00	Flavonoids	100.00	Flavones	100.00
4-Ethylcatechol	Shikimates and Phenylpropanoids	76.80	Phenylethanoids (C6–C2)	9.60	Phenylethanoids	11.20
9,10-Dihydroxyanthracene	Polyketides	91.50	Naphthalenes	98.10	Naphthoquinones	85.20
4-Heptyloxyphenol	Shikimates and Phenylpropanoids	24.30	Phenolic acids (C6–C1)	2.90	Hydrocarbons	3.10

**Table A4:** Discordant superclass assignments in Cascara (3/93 compounds; overall concordance 96.8%).

Component	Validated Superclass	Predicted Superclass	Acc.%
4-Ethylcatechol	Benzenoids	Phenylethanoids (C6–C2)	9.6
9,10-Dihydroxyanthracene	Polycyclic aromatic polyketides	Naphthalenes	98.10
4-Heptyloxyphenol	Aromatic polyketides	Phenolic acids (C6–C1)	2.9

The few discordant cases reflect borderline structural features. For instance, 4-ethylcatechol was misclassified as a phenylethanoid due to the presence of a short C2 side chain, although it lacks the typical conjugated system of this class. Similarly, 9,10-dihydroxyanthracene was predicted as a naphthalene, likely because the model emphasized the fused aromatic scaffold rather than the oxidized anthracene core. Finally, 4-heptyloxyphenol was assigned to phenolic acids despite the absence of a carboxyl group, a misclassification probably arising from underrepresentation of long-chain alkylphenols in the training dataset.

### Appendix A.3. Frangula

For Frangula, Table A5 lists all identified metabolites with validated superclass assignments, while Table A6 provides the hierarchical classification with model-derived confidence scores. Table A7 highlights the few discordant cases, which are discussed further below. For full-precision *m*/*z* values (reported to four decimal places) together with the corresponding retention times (RT) and mass errors (Δ, ppm), readers are referred to Supplementary Table S1.

**Table A5:** Frangula—Identified metabolites with validated *Superclass*. Molecular weights are rounded to two decimals.

Component	Formula	Molecular Weight	Superclass
Pavine	C_20_H_23_NO_4_	341.16	Tyrosine alkaloids
Emodin	C_15_H_10_O_5_	270.05	Polycyclic aromatic polyketides
Liquiritin	C_21_H_22_O_9_	418.13	Flavonoids
Genistein	C_15_H_10_O_5_	270.05	Isoflavonoids
Glucofrangulin A	C_27_H_30_O_14_	578.17	Polycyclic aromatic polyketides
Unknown compound	–	528.19	–
Unknown compound	–	484.16	–
Oleuropein	C_25_H_32_O_13_	540.19	Monoterpenoids
Demethyloleuropein	C_24_H_30_O_13_	526.17	Monoterpenoids
Unknown compound	–	510.18	–
Unknown compound	–	496.16	–
Glucofrangulin B	C_26_H_28_O_14_	564.15	Polycyclic aromatic polyketides
Isoliquiritigenin	C_15_H_12_O_4_	256.07	Flavonoids
5,6-O-β-D-diglucopyranosylangelicin	C_23_H_26_O_15_	542.13	Coumarins
Kaempferol	C_15_H_10_O_6_	286.05	Flavonoids
Frangulin A	C_21_H_20_O_9_	416.11	Polycyclic aromatic polyketides
Frangulin B	C_20_H_18_O_9_	402.10	Polycyclic aromatic polyketides
Lespedin	C_27_H_30_O_14_	578.17	Flavonoids
Phloretin	C_15_H_14_O_5_	274.08	Flavonoids
2-Ethyl-9,10-anthraquinone	C_16_H_12_O_2_	236.08	Polycyclic aromatic polyketides
Neohesperidin dihydrochalcone	C_28_H_36_O_15_	612.21	Flavonoids
Nepodin	C_13_H_12_O_3_	216.08	Naphthalenes
Daidzein	C_15_H_10_O_4_	254.06	Isoflavonoids
Piceatannol	C_14_H_12_O_4_	244.07	Stilbenoids
trans-Caffeic acid	C_9_H_8_O_4_	180.04	Phenylpropanoids (C6–C3)
6-Methoxyflavonol	C_16_H_12_O_4_	268.07	Flavonoids
Gossypetin 3-sophoroside-8-glucoside	C_33_H_40_O_23_	804.20	Flavonoids
3-Caffeoylquinic acid	C_16_H_18_O_9_	354.09	Phenylpropanoids (C6–C3)
Laricitrin 3,7,$5^{\prime }$-triglucoside	C_34_H_42_O_23_	818.21	Flavonoids
Patuletin 3-($4^{\prime \prime }$-acetylrhamnoside)-7-($2^{\prime \prime \prime }$,$4^{\prime \prime \prime }$-diacetylrhamnoside)	C_34_H_38_O_19_	750.20	Flavonoids
Acaciin	C_28_H_32_O_14_	592.18	Flavonoids
Pinocembrin	C_15_H_12_O_4_	256.07	Flavonoids
Quercitrin	C_21_H_20_O_11_	448.10	Flavonoids
Isoschaftoside	C_26_H_28_O_14_	564.15	Flavonoids
Methyl chlorogenate	C_17_H_20_O_9_	368.11	Phenylpropanoids (C6–C3)
Caffeic acid 3-glucoside	C_15_H_18_O_9_	342.10	Phenylpropanoids (C6–C3)
Luteolin	C_15_H_10_O_6_	286.05	Flavonoids
Kaempferide	C_16_H_12_O_6_	300.06	Flavonoids
Methyl 4-methoxycinnamate	C_11_H_12_O_3_	192.08	Phenylpropanoids (C6–C3)
Narirutin	C_27_H_32_O_14_	580.18	Flavonoids
Loganin	C_17_H_26_O_10_	390.15	Monoterpenoids
Apiin	C_26_H_28_O_14_	564.15	Flavonoids
Taxifolin	C_15_H_12_O_7_	304.06	Flavonoids
Afzelechin	C_15_H_14_O_5_	274.08	Flavonoids
Diosmetin 7-O-β-D-glucoside	C_22_H_22_O_11_	462.12	Flavonoids
isorhamnetin 3-O-alpha-L-[$6^{⁗}$-p-coumaroyl-β-D-glucopyranosyl-(1→2)-rhamnopyranoside]	C_37_H_38_O_18_	770.21	Flavonoids
Oleic acid	C_18_H_34_O_2_	282.26	Fatty acids and conjugates
$3^{\prime }$,$4^{\prime }$,5,7-Tetramethyldihydroquercetin	C_19_H_20_O_7_	360.12	Flavonoids
3-Methoxynobiletin	C_22_H_24_O_9_	432.14	Flavonoids
Epigallocatechin-(4β→8)-$4^{\prime }$-O-methylgallocatechin	C_31_H_28_O_14_	624.15	Flavonoids
Primuletin	C_15_H_10_O_3_	238.06	Flavonoids
$2^{\prime \prime }$-Acetylpaeonoside	C_29_H_32_O_17_	652.16	Flavonoids
2,3-Dihydro-$4^{\prime }$,$4^{\prime \prime \prime }$-di-O-methylamentoflavone	C_32_H_24_O_10_	568.14	Flavonoids
Piceid	C_20_H_22_O_8_	390.13	Stilbenoids
Vicenin-2	C_27_H_30_O_15_	594.16	Flavonoids
Chrysoeriol 7-($2^{\prime \prime \prime }$-feruloylglucuronosyl)-(1→2)-glucuronide	C_38_H_36_O_21_	828.17	Flavonoids
$4^{\prime }$,5,7-Trimethoxyflavone	C_18_H_16_O_5_	312.10	Flavonoids
Catechin	C_15_H_14_O_6_	290.08	Flavonoids
Rutin	C_27_H_30_O_16_	610.15	Flavonoids
Sakuranetin	C_16_H_14_O_5_	286.08	Flavonoids
Hesperetin	C_16_H_14_O_6_	302.08	Flavonoids
Flavanol	C_15_H_14_O_6_	290.08	Flavonoids
Hispidulin	C_16_H_12_O_6_	300.06	Flavonoids
7-Methoxyflavone	C_16_H_12_O_3_	252.08	Flavonoids
Diosmin	C_28_H_32_O_15_	608.17	Flavonoids
Schaftoside	C_26_H_28_O_14_	564.15	Flavonoids
(E)-4-Methoxycinnamic acid	C_10_H_10_O_3_	178.06	Phenylpropanoids (C6–C3)
Isoformononetin	C_16_H_12_O_4_	268.07	Isoflavonoids
Isoquercetin	C_21_H_20_O_12_	464.10	Flavonoids
Malonyldaidzin	C_24_H_22_O_12_	502.11	Isoflavonoids
Quercetin 3-O-xylosyl-rutinoside	C_32_H_38_O_20_	742.20	Flavonoids
Liquiritin apioside	C_26_H_30_O_13_	550.17	Flavonoids
Isovitexin 7-O-[feruloyl]-glucoside	C_37_H_38_O_18_	770.20	Flavonoids
Isoquercitrin	C_21_H_20_O_12_	464.10	Flavonoids
1,2,4-Trihydroxyanthraquinone	C_14_H_8_O_5_	256.04	Polycyclic aromatic polyketides
Kaempferol 3-($6^{\prime \prime \prime }$-rhamnosyl-$2^{\prime \prime \prime }$-(6-malyl-glucosyl)-glucoside)	C_37_H_44_O_24_	872.22	Flavonoids
Quercetin-3-O-($6^{\prime \prime \prime }$-trans-p-coumaroyl-$2^{\prime \prime }$-glucosyl)rhamnoside	C_36_H_36_O_18_	756.19	Flavonoids
1,2,8-Trihydroxyanthraquinone	C_14_H_8_O_5_	256.04	Polycyclic aromatic polyketides
4-Heptyloxyphenol	C_13_H_20_O_2_	208.15	Aromatic polyketides
5,7-DIHYDROXY-$3^{\prime }$,$4^{\prime }$,$5^{\prime }$-TRIMETHOXYFLAVANONE	C_18_H_18_O_7_	346.11	Flavonoids
7-[(6-Deoxy-α-L-mannopyranosyl)oxy]-2-(4-hydroxyphenyl)-4-oxo-4H-chromen-3-yl 6-O-(6-deoxy-α-L-mannopyranosyl)-β-D-glucopyranoside	C_33_H_40_O_18_	724.22	Flavonoids
4-Hydroxy-3-methyl-9,10-dioxo-9,10-dihydro-2-anthracenyl 6-O-acetyl-2-O-(6-deoxy-α-L-mannopyranosyl)-β-D-glucopyranoside	C_29_H_32_O_14_	604.18	Polycyclic aromatic polyketides
5-Hydroxy-3,6,7,8,$3^{\prime }$,$4^{\prime }$-hexamethoxyflavone	C_21_H_22_O_9_	418.13	Flavonoids

**Table A6:** Frangula—Hierarchical classification of identified metabolites with model accuracy expressed as percentage.

Component	Pathway	Acc.%	Superclass	Acc.%	Class	Acc.%
Pavine	Tyrosine alkaloids	99.90	Tyrosine alkaloids	99.90	Isoquinoline alkaloids	88.80
Emodin	Polycyclic aromatic polyketides	99.90	Polycyclic aromatic polyketides	99.90	Anthraquinones and anthrones	99.90
Liquiritin	Flavonoids	99.90	Flavonoids	99.90	Flavanones	99.90
Genistein	Isoflavonoids	99.90	Isoflavonoids	99.90	Isoflavones	99.80
Glucofrangulin A	Polycyclic aromatic polyketides	99.90	Polycyclic aromatic polyketides	99.90	Anthraquinones and anthrones	99.90
Unknown compound	–		–		–	
Unknown compound	–		–		–	
Oleuropein	Monoterpenoids	99.90	Monoterpenoids	99.90	Secoiridoid monoterpenoids	100.00
Demethyloleuropein	Monoterpenoids	99.90	Monoterpenoids	99.90	Secoiridoid monoterpenoids	100.00
Unknown compound	–		–		–	
Unknown compound	–		–		–	
Glucofrangulin B	Polycyclic aromatic polyketides	99.90	Polycyclic aromatic polyketides	99.90	Anthraquinones and anthrones	99.90
Isoliquiritigenin	Flavonoids	99.90	Flavonoids	99.90	Chalcones	100.00
5,6-O-β-D-diglucopyranosylangelicin	Coumarins	99.90	Coumarins	99.90	Furocoumarins	99.90
Kaempferol	Flavonoids	99.90	Flavonoids	99.90	Flavonols	99.90
Frangulin A	Polycyclic aromatic polyketides	99.90	Polycyclic aromatic polyketides	99.90	Anthraquinones and anthrones	99.90
Frangulin B	Polycyclic aromatic polyketides	99.90	Polycyclic aromatic polyketides	99.90	Anthraquinones and anthrones	99.90
Lespedin	Flavonoids	99.90	Flavonoids	99.90	Flavonols	99.90
Phloretin	Flavonoids	99.90	Flavonoids	99.90	Chalcones	99.90
2-Ethyl-9,10-anthraquinone	Polycyclic aromatic polyketides	99.90	Polycyclic aromatic polyketides	99.90	Anthraquinones and anthrones	99.90
Neohesperidin dihydrochalcone	Flavonoids	99.90	Flavonoids	99.90	Chalcones	99.90
Nepodin	Naphthalenes	99.90	Naphthalenes	99.90	Naphthalenes and derivatives	99.90
Daidzein	Isoflavonoids	99.90	Isoflavonoids	99.90	Isoflavones	99.90
Piceatannol	Stilbenoids	99.90	Stilbenoids	99.90	Monomeric stilbenes	98.70
trans-caffeic acid	Phenylpropanoids (C6–C3)	96.20	Phenylpropanoids (C6–C3)	96.20	Cinnamic acids and derivatives	98.20
6-Methoxyflavonol	Flavonoids	99.90	Flavonoids	99.90	Flavonols	99.90
Gossypetin 3-sophoroside-8-glucoside	Flavonoids	99.90	Flavonoids	99.90	Flavonols	99.90
3-Caffeoylquinic acid	Phenylpropanoids (C6–C3)	97.40	Phenylpropanoids (C6–C3)	97.10	Cinnamic acids and derivatives	99.90
Laricitrin 3,7,$5^{\prime }$-triglucoside	Flavonoids	99.90	Flavonoids	99.90	Flavonols	99.90
Patuletin 3-($4^{\prime \prime }$-acetylrhamnoside)-7-($2^{\prime \prime \prime }$,$4^{\prime \prime \prime }$-diacetylrhamnoside)	Flavonoids	99.90	Flavonoids	99.90	Flavonols	99.90
Acaciin	Flavonoids	99.90	Flavonoids	99.90	Flavones	99.90
Pinocembrin	Flavonoids	99.90	Flavonoids	99.90	Flavanones	99.70
Quercitrin	Flavonoids	99.90	Flavonoids	99.90	Flavonols	99.90
Isoschaftoside	Flavonoids	99.90	Flavonoids	99.90	Flavones	99.90
Methyl chlorogenate	Phenylpropanoids (C6–C3)	98.50	Phenylpropanoids (C6–C3)	98.50	Cinnamic acids and derivatives	99.50
Caffeic acid 3-glucoside	Phenylpropanoids (C6–C3)	98.10	Phenylpropanoids (C6–C3)	98.10	Cinnamic acids and derivatives	99.00
Luteolin	Flavonoids	99.90	Flavonoids	99.90	Flavones	99.90
Kaempferide	Flavonoids	99.90	Flavonoids	99.90	Flavones	99.90
Methyl 4-methoxycinnamate	Phenylpropanoids (C6–C3)	98.50	Phenylpropanoids (C6–C3)	98.50	Cinnamic acids and derivatives	98.00
Narirutin	Flavonoids	99.90	Flavonoids	99.90	Flavanones	99.90
Loganin	Monoterpenoids	99.90	Monoterpenoids	99.90	Iridoids monoterpenoids	99.90
Apiin	Flavonoids	99.90	Flavonoids	99.90	Flavones	99.90
Taxifolin	Flavonoids	99.90	Flavonoids	99.90	Dihydroflavonols	99.90
Afzelechin	Flavonoids	99.90	Flavonoids	99.90	Flavan-3-ols	99.80
Diosmetin 7-O-β-D-glucoside	Flavonoids	99.90	Flavonoids	99.90	Flavones	99.90
isorhamnetin 3-O-α-L-[$6^{⁗}$-p-coumaroyl-β-D-glucopyranosyl-(1→2)-rhamnopyranoside]	Flavonoids	100.00	Flavonoids	100.00	Flavonols	99.90
Oleic acid	Fatty Acids and Conjugates	97.20	Fatty Acids and Conjugates	97.20	Unsaturated fatty acids	90.30
$3^{\prime }$,$4^{\prime }$,5,7-tetramethyldihydroquercetin	Flavonoids	99.90	Flavonoids	99.90	Dihydroflavonols	99.70
3-Methoxynobiletin	Flavonoids	99.90	Flavonoids	99.90	Flavonols	99.90
epigallocatechin-(4β→8)-$4^{\prime }$-O-methylgallocatechin	Flavonoids	99.90	Flavonoids	99.90	Proanthocyanins	99.60
Primuletin	Flavonoids	99.90	Flavonoids	99.90	Flavones	99.90
$2^{\prime \prime }$-acetylpaeonoside	Flavonoids	99.90	Flavonoids	99.90	Flavonols	99.90
2,3-dihydro-$4^{\prime }$,$4^{\prime \prime \prime }$-di-O-methylamentoflavone	Flavonoids	100.00	Flavonoids	100.00	Flavanones;Flavones	99.40
Piceid	Stilbenoids	99.90	Stilbenoids	99.90	Monomeric stilbenes	98.90
vicenin 2	Flavonoids	99.90	Flavonoids	99.90	Flavones	99.90
Chrysoeriol 7- ($2^{\prime \prime \prime }$-feruloylglucuronosyl) - (1→2) -glucuronide	Flavonoids	99.90	Flavonoids	99.90	Flavones	99.90
$4^{\prime }$,5,7-TRIMETHOXYFLAVONE	Flavonoids	99.90	Flavonoids	99.90	Flavones	99.90
Catechin	Flavonoids	99.90	Flavonoids	99.90	Flavan-3-ols	99.80
Rutin	Flavonoids	99.90	Flavonoids	99.90	Flavonols	99.90
Sakuranetin	Flavonoids	99.90	Flavonoids	99.90	Flavanones	99.90
Hesperetin	Flavonoids	99.90	Flavonoids	99.90	Flavanones	99.80
Flavanol	Flavonoids	99.90	Flavonoids	99.90	Flavanones;Flavans	74.50
Hispidulin	Flavonoids	99.90	Flavonoids	99.90	Flavones	99.90
7-methoxyflavone	Flavonoids	99.90	Flavonoids	99.90	Flavones	99.90
Diosmin	Flavonoids	99.90	Flavonoids	99.90	Flavones	99.90
schaftoside	Flavonoids	99.90	Flavonoids	99.90	Flavones	99.90
(E)-4-Methoxycinnamic acid	Phenylpropanoids (C6–C3)	97.90	Phenylpropanoids (C6–C3)	97.90	Cinnamic acids and derivatives	97.30
isoformononetin	Isoflavonoids	99.90	Isoflavonoids	99.90	Isoflavones	99.90
isoquercetin	Flavonoids	99.90	Flavonoids	99.90	Flavonols	99.90
malonyldaidzin	Isoflavonoids	99.90	Isoflavonoids	99.90	Isoflavones	99.90
Quercetin 3-O-xylosyl-rutinoside	Flavonoids	99.90	Flavonoids	99.90	Flavonols	99.90
LIQUIRITIN APIOSIDE	Flavonoids	99.90	Flavonoids	99.90	Flavanones	99.90
isovitexin 7-O-[feruloyl]-glucoside	Flavonoids	100.00	Flavonoids	100.00	Flavones	99.90
Isoquercitrin	Flavonoids	99.90	Flavonoids	99.90	Flavonols	99.90
1,2,4-Trihydroxyanthraquinone	Polycyclic aromatic polyketides	99.90	Polycyclic aromatic polyketides	99.90	Anthraquinones and anthrones	99.90
Kaempferol 3- ($6^{\prime \prime \prime }$-rhamnosyl-$2^{\prime \prime \prime }$- (6-malyl-glucosyl) -glucoside)	Flavonoids	99.90	Flavonoids	99.90	Flavonols	99.90
Quercetin-3-O-($6^{\prime \prime \prime }$-trans-p-coumaroyl-$2^{\prime \prime }$-glucosyl)rhamnoside	Flavonoids	100.00	Flavonoids	100.00	Flavonols	99.90
1,2,8-Trihydroxyanthraquinone	Polycyclic aromatic polyketides	99.90	Polycyclic aromatic polyketides	99.90	Anthraquinones and anthrones	99.80
4-Heptyloxyphenol	Phenolic acids (C6–C1)	2.90	Phenolic acids (C6–C1)	2.90	Hydrocarbons	3.00
5,7-DIHYDROXY-$3^{\prime }$,$4^{\prime }$,$5^{\prime }$-TRIMETHOXYFLAVANONE	Anthranilic acid alkaloids	1.30	Anthranilic acid alkaloids	1.30	Pyridine alkaloids	70.70
7-[(6-Deoxy-α-L-mannopyranosyl)oxy]-2-(4-hydroxyphenyl)-4-oxo-4H-chromen-3-yl 6-O-(6-deoxy-alpha-L-mannopyranosyl)-β-D-glucopyranoside	-					
4-Hydroxy-3-methyl-9,10-dioxo-9,10-dihydro-2-anthracenyl 6-O-acetyl-2-O-(6-deoxy-α-L-mannopyranosyl)-β-D-glucopyranoside	–		–		–	
5-hydroxy-3,6,7,8,$3^{\prime }$,$4^{\prime }$-hexamethoxyflavone	–		–		–	

**Table A7:** Discordant and unassigned superclass assignments in Frangula (5/83 compounds; overall concordance 94.0%).

Component	Validated Superclass	Predicted Superclass	Acc.%
4-Heptyloxyphenol	Aromatic polyketides	Phenolic acids (C6–C1)	2.9
5,7-DIHYDROXY-$3^{\prime }$,$4^{\prime }$,$5^{\prime }$-TRIMETHOXYFLAVANONE	Flavonoids	Anthranilic acid alkaloids	1.3
7-[(6-Deoxy-α-L-mannopyranosyl)oxy]-2-(4-hydroxyphenyl)-4-oxo-4H-chromen-3-yl 6-O-(6-deoxy-alpha-L-mannopyranosyl)-β-D-glucopyranoside	Flavonoids	–	–
4-Hydroxy-3-methyl-9,10-dioxo-9,10-dihydro-2-anthracenyl 6-O-acetyl-2-O-(6-deoxy-α-L-mannopyranosyl)-β-D-glucopyranoside	Anthraquinones and anthrones	–	–
5-hydroxy-3,6,7,8,$3^{\prime }$,$4^{\prime }$-hexamethoxyflavone	Flavonoids	–	–

The few discordant cases reflect borderline structural features or gaps in the training data. For instance, 4-heptyloxyphenol was predicted as a phenolic acid despite lacking a carboxyl group, likely because the long alkyl side chain was poorly represented in training examples. Similarly, 5,7-dihydroxy-$3^{\prime }$,$4^{\prime }$,$5^{\prime }$-trimethoxyflavanone, a typical flavonoid, was misclassified as an anthranilic acid alkaloid, probably due to the presence of multiple methoxy substituents mimicking alkaloid-like motifs. In addition, three compounds (a glycosylated flavone derivative, an anthracene glycoside, and a highly methoxylated flavone) were not assigned at all, reflecting either insufficient representation of complex glycosylated structures or ambiguous fragmentation patterns. These cases underline typical boundary conditions in automated annotation, but were readily resolved by expert review without altering the main compositional trends.

### Appendix A.4. Rhubarb

For Rhubarb, Table A8 lists all identified metabolites with validated superclass assignments, while Table A9 provides the hierarchical classification with model-derived confidence scores. Table A10 highlights the few discordant cases, which are discussed further below. For full-precision *m*/*z* values (reported to four decimal places) together with the corresponding retention times (RT) and mass errors (Δ, ppm), readers are referred to Supplementary Table S1.

**Table A8:** Rhubarb—Identified metabolites with validated *Superclass*. Molecular weights are rounded to two decimals.

Component	Formula	Molecular Weight	Superclass
Emodin	C_15_H_10_O_5_	270.05	Polycyclic aromatic polyketides
Piceatannol	C_14_H_12_O_4_	244.07	Stilbenoids
Rhein	C_15_H_8_O_6_	284.03	Polycyclic aromatic polyketides
Daidzein	C_15_H_10_O_4_	254.06	Isoflavonoids
Catechin	C_15_H_14_O_6_	290.08	Flavonoids
Rhein-8-glucoside	C_21_H_18_O_11_	446.08	Polycyclic aromatic polyketides
Genistein	C_15_H_10_O_5_	270.05	Isoflavonoids
(2Z)-6-hydroxy-2-[(4-hydroxy-3-methoxyphenyl) methylidene]-2,3-dihydro-1-benzofuran-3-one	C_16_H_12_O_5_	284.07	Flavonoids
3-O-Methylquercetintetraacetate	C_24_H_20_O_11_	484.10	Flavonoids
(+)-Catechin 3-O-gallate	C_22_H_18_O_10_	442.09	Flavonoids
Torachrysone 8-O-β-D-glucoside	C_20_H_24_O_9_	408.14	Naphthalenes
spectaflavoside A	C_46_H_42_O_22_	946.22	Flavonoids
1,4-Dihydroxy-5,8-bis(p-toluidino)anthraquinone	C_28_H_22_N_2_O_4_	450.16	Polycyclic aromatic polyketides
5-Acetonyl-7-hydroxy-2-methylchromone	C_13_H_12_O_4_	232.07	Chromanes
aloesin	C_19_H_22_O_9_	394.13	Chromanes
Protocatechuic aldehyde	C_7_H_6_O_3_	138.03	Phenolic acids (C6–C1)
Kaempferol	C_15_H_10_O_6_	286.05	Flavonoids
Limocitrol 3- [ α-L-arabinopyranosyl- (1→3) [ galactosyl- (1→6) ] -galactoside ]	C_35_H_44_O_23_	832.23	Flavonoids
Irilone	C_16_H_10_O_6_	298.05	Isoflavonoids
Afzelechin	C_15_H_14_O_5_	274.08	Flavonoids
Cassialoin	C_21_H_22_O_9_	418.13	Polycyclic aromatic polyketides
Isoliquiritigenin	C_15_H_12_O_4_	256.07	Flavonoids
Vitexin	C_21_H_20_O_10_	432.11	Flavonoids
Eriodictyol	C_15_H_12_O_6_	288.06	Flavonoids
$4^{\prime }$,$4^{\prime \prime }$-DIMETHYLEPIGALLOCATECHIN GALLATE	C_24_H_22_O_11_	486.12	Flavonoids
Resveratrol	C_14_H_12_O_3_	228.08	Stilbenoids
Chrysin	C_15_H_10_O_4_	254.06	Flavonoids
Resveratrol 3-O-glucoside	C_20_H_22_O_8_	390.13	Stilbenoids
Kaempferol 3-O-glucosyl-rhamnosyl-galactoside	C_33_H_40_O_20_	756.21	Flavonoids
1,2,4-Trihydroxyanthraquinone	C_14_H_8_O_5_	256.04	Polycyclic aromatic polyketides
Gallic acid	C_7_H_6_O_5_	170.02	Phenolic acids (C6–C1)
aescuflavoside	C_38_H_48_O_25_	904.25	Flavonoids
Hispidulin	C_16_H_12_O_6_	300.06	Flavonoids
$3^{\prime }$,$4^{\prime }$,7-Trihydroxyflavanone	C_15_H_12_O_5_	272.07	Flavonoids
Apigenin	C_15_H_10_O_5_	270.05	Flavonoids
Pinocembrin	C_15_H_12_O_4_	256.07	Flavonoids
HEXAMETHYLQUERCETAGETIN	C_21_H_22_O_8_	402.13	Flavonoids
3-Hydroxyflavone	C_15_H_10_O_3_	238.06	Flavonoids
Primuletin	C_15_H_10_O_3_	238.06	Flavonoids
Quercetin 3-sambubioside-$3^{\prime }$-glucoside	C_32_H_38_O_21_	758.19	Flavonoids
Phloretin	C_15_H_14_O_5_	274.08	Flavonoids
3-hydroxyflavanone	C_15_H_12_O_3_	240.08	Flavonoids
Taxifolin	C_15_H_12_O_7_	304.06	Flavonoids
Quercetin 3-O-xylosyl-rutinoside	C_32_H_38_O_20_	742.19	Flavonoids
Asebogenin	C_16_H_16_O_5_	288.10	Flavonoids
Formononetin	C_16_H_12_O_4_	268.07	Isoflavonoids
calabricoside A	C_32_H_38_O_20_	742.20	Flavonoids
tricin 7-O-($6^{\prime \prime }$-O-malonyl)-β-D-glucopyranoside	C_26_H_26_O_15_	578.13	Flavonoids
Acacetin	C_16_H_12_O_5_	284.07	Flavonoids
chrysoobtusin	C_19_H_18_O_7_	358.11	Polycyclic aromatic polyketides
1-AMINO-4-BENZAMIDOANTHRAQUINONE	C_21_H_14_N_2_O_3_	342.10	Polycyclic aromatic polyketides
Quercetin 3-rutinoside-7-glucuronide	C_33_H_38_O_22_	786.19	Flavonoids
Kaempferol 3-O-glucosyl-rhamnosyl-galactoside	C_33_H_40_O_20_	756.21	Flavonoids
Syringetin-3-glucoside	C_23_H_24_O_13_	508.12	Flavonoids
quercetin 3-O-(3-O-p-coumaroyl, 6-O-feruloyl)-glucoside	C_40_H_34_O_17_	786.18	Flavonoids
1-Formyl-4-hydroxyanthraquinone	C_15_H_8_O_4_	252.04	Polycyclic aromatic polyketides
quercetin 3-O-gentiobioside-7-O-rhamnoside	C_33_H_40_O_21_	772.20	Flavonoids
kaempferol 3-O-gentiobioside-7-O-rhamnoside	C_33_H_40_O_20_	756.21	Flavonoids
Quercetin 3-glucosyl-(1→3)-rhamnosyl-(1→6)-galactoside	C_33_H_40_O_21_	772.21	Flavonoids
Quercetin 3-rutinoside-7,$3^{\prime }$-diglucoside	C_39_H_50_O_26_	934.26	Flavonoids
Ononin	C_22_H_22_O_9_	430.13	Isoflavonoids
Tetramethylscutellarein	C_19_H_18_O_6_	342.11	Flavonoids
Carnosol	C_20_H_26_O_4_	330.18	Diterpenoids
KAEMPFEROL-3-O-($6^{\prime \prime \prime }$-TRANS-P-COUMAROYL-$2^{\prime \prime }$-GLUCOSYL)RHAMNOSIDE	C_36_H_36_O_17_	740.20	Flavonoids
aromadendrin	C_15_H_12_O_6_	288.06	Flavonoids
Biochanin A	C_16_H_12_O_5_	284.07	Isoflavonoids
Glycitein	C_16_H_12_O_5_	284.07	Flavonoids
(-)-L-Chicoric acid	C_22_H_18_O_12_	474.08	Phenylpropanoids (C6–C3)
Scutellarin	C_21_H_18_O_12_	462.08	Flavonoids
Ellagic acid	C_14_H_6_O_8_	302.01	Phenolic acids (C6–C1)
Myricetin	C_15_H_10_O_8_	318.04	Flavonoids
Luteolinidin	C_15_H_11_O_5_	271.06	Flavonoids
(E)-Ferulic acid	C_10_H_10_O_4_	194.06	Phenylpropanoids (C6–C3)
quercetin 3-O-sophoroside-7-O-rhamnoside	C_33_H_40_O_21_	772.21	Flavonoids
Glycitin	C_22_H_22_O_10_	446.12	Isoflavonoids
4-Heptyloxyphenol	C_13_H_20_O_2_	208.15	Aromatic polyketides
$4^{\prime }$-HYDROXYFLAVONE	C_15_H_10_O_3_	238.06	Flavonoids
$2^{\prime \prime }$,$6^{\prime \prime }$-O-diacetyloninin	C_26_H_26_O_11_	514.15	Isoflavonoids
ANTRAQUINONE DERIVATIVE	C_14_H_8_O_4_	240.04	Polycyclic aromatic polyketides
6-(β-D-Glucopyranuronosyloxy)-5,7-dihydroxy-2-(4-hydroxyphenyl)-4-oxo-4H-chromen-8-yl β-D-glucopyranosiduronic acid	C_27_H_26_O_19_	654.11	Flavonoids
2-(3,4-Dihydroxyphenyl)-7- (β-D-glucopyranosyloxy)-5-hydroxy-4-oxo-4H-chromen-3-yl 6-deoxy-α-L-mannopyranosyl-(1→2)-[6-deoxy-α-L-mannopyranosyl-(1→6)]-β-D-galactopyranoside	C_39_H_50_O_25_	918.26	Flavonoids
5,7-Dihydroxy-2-(4-hydroxy-3-methoxyphenyl) -4-oxo-4H-chromen-3-yl β-D-glucopyranosyl-(1→3)-6-deoxy-α-L-mannopyranosyl-(1→6)-β-D-glucopyranoside	C_34_H_42_O_21_	786.22	Flavonoids
Kaempferol 7-methyl ether 3- (6- (E) -3,5- dimethoxy-4-hydroxycinnamoylglucosyl) - (1→2) - [ rhamnosyl- (1→6) -glucoside ]	C_45_H_52_O_24_	976.29	Flavonoids

**Table A9:** Rhubarb—Hierarchical classification of identified metabolites with model accuracy expressed as percentage.

Component	Pathway	Acc.%	Superclass	Acc.%	Class	Acc.%
Emodin	Polyketides	99.90	Polycyclic aromatic polyketides	99.90	Anthraquinones and anthrones	99.90
Piceatannol	Shikimates and Phenylpropanoids	99.80	Stilbenoids	99.90	Monomeric stilbenes	98.70
Rhein	Polyketides	99.90	Polycyclic aromatic polyketides	99.90	Anthraquinones and anthrones	99.90
Daidzein	Shikimates and Phenylpropanoids	99.90	Isoflavonoids	99.90	Isoflavones	99.90
Catechin	Shikimates and Phenylpropanoids	99.80	Flavonoids	99.90	Flavan-3-ols	99.80
Rhein-8-glucoside	Polyketides	99.90	Polycyclic aromatic polyketides	99.90	Anthraquinones and anthrones	99.90
Genistein	Shikimates and Phenylpropanoids	99.80	Isoflavonoids	99.90	Isoflavones	99.80
(2Z)-6-hydroxy-2-[(4-hydroxy-3-methoxyphenyl)methylidene]-2,3-dihydro-1-benzofuran-3-one	Shikimates and Phenylpropanoids	99.80	Flavonoids	99.90	Aurones	99.90
3-O-Methylquercetintetraacetate	Shikimates and Phenylpropanoids	99.30	Flavonoids	99.90	Flavonols	99.90
(+)-Catechin 3-O-gallate	Shikimates and Phenylpropanoids	99.60	Flavonoids	99.90	Flavan-3-ols	99.90
Torachrysone 8-O-β-D-glucoside	Polyketides	99.00	Naphthalenes	99.80	Naphthalenes and derivatives	99.90
spectaflavoside A	Shikimates and Phenylpropanoids	100.00	Flavonoids	99.90	Flavonols	100.00
1,4-Dihydroxy-5,8-bis(p-toluidino)anthraquinone	Polyketides	99.90	Polycyclic aromatic polyketides	99.90	Anthraquinones and anthrones	93.40
5-Acetonyl-7-hydroxy-2-methylchromone	Polyketides	99.60	Chromanes	99.90	Chromones	99.90
aloesin	Polyketides	99.80	Chromanes	99.90	Chromones	99.90
Protocatechuic aldehyde	Terpenoids	52.00	Phenolic acids (C6–C1)	83.80	Simple phenolic acids	45.00
Kaempferol	Shikimates and Phenylpropanoids	99.90	Flavonoids	99.90	Flavonols	99.90
Limocitrol 3- [ α-L-arabinopyranosyl- (1→3) [ galactosyl- (1→6) ] -galactoside ]	Shikimates and Phenylpropanoids	99.90	Flavonoids	99.90	Flavonols	99.90
Irilone	Shikimates and Phenylpropanoids	99.90	Isoflavonoids	99.90	Isoflavones	99.70
Afzelechin	Shikimates and Phenylpropanoids	99.80	Flavonoids	99.90	Flavan-3-ols	99.80
Cassialoin	Polyketides	99.90	Polycyclic aromatic polyketides	99.90	Anthraquinones and anthrones	99.90
Isoliquiritigenin	Shikimates and Phenylpropanoids	99.90	Flavonoids	99.90	Chalcones	100.00
Vitexin	Shikimates and Phenylpropanoids	99.90	Flavonoids	99.90	Flavones	99.90
Eriodictyol	Shikimates and Phenylpropanoids	99.90	Flavonoids	99.90	Flavanones	99.70
$4^{\prime }$,$4^{\prime \prime }$-DIMETHYL EPIGALLOCATECHIN GALLATE	Shikimates and Phenylpropanoids	99.90	Flavonoids	99.90	Flavan-3-ols	99.70
Resveratrol	Shikimates and Phenylpropanoids	99.80	Stilbenoids	99.90	Monomeric stilbenes	98.00
Chrysin	Shikimates and Phenylpropanoids	99.40	Flavonoids	99.90	Flavones	100.00
Resveratrol 3-O-glucoside	Shikimates and Phenylpropanoids	99.90	Stilbenoids	99.90	Monomeric stilbenes	98.90
Kaempferol 3-O-glucosyl-rhamnosyl-galactoside	Shikimates and Phenylpropanoids	99.90	Flavonoids	99.90	Flavonols	99.90
1,2,4-Trihydroxyanthraquinone	Polyketides	99.90	Polycyclic aromatic polyketides	99.90	Anthraquinones and anthrones	99.90
Gallic acid	Shikimates and Phenylpropanoids	97.90	Phenolic acids (C6–C1)	97.90	Simple phenolic acids	97.00
aescuflavoside	Shikimates and Phenylpropanoids	99.90	Flavonoids	99.90	Flavonols	99.90
Hispidulin	Shikimates and Phenylpropanoids	99.90	Flavonoids	99.90	Flavones	99.90
$3^{\prime }$,$4^{\prime }$,7-Trihydroxyflavanone	Shikimates and Phenylpropanoids	99.90	Flavonoids	99.90	Flavanones	99.80
Apigenin	Shikimates and Phenylpropanoids	99.90	Flavonoids	99.90	Flavones	99.90
Pinocembrin	Shikimates and Phenylpropanoids	99.90	Flavonoids	99.90	Flavanones	99.70
HEXAMETHYL QUERCETAGETIN	Shikimates and Phenylpropanoids	99.90	Flavonoids	99.90	Flavonols	99.90
3-Hydroxyflavone	Shikimates and Phenylpropanoids	99.80	Flavonoids	99.90	Flavonols	99.90
Primuletin	Shikimates and Phenylpropanoids	98.60	Flavonoids	99.90	Flavones	99.90
Quercetin 3-sambubioside-$3^{\prime }$-glucoside	Shikimates and Phenylpropanoids	99.90	Flavonoids	99.90	Flavonols	99.90
Phloretin	Shikimates and Phenylpropanoids	99.10	Flavonoids	99.90	Chalcones	99.90
3-hydroxyflavanone	Shikimates and Phenylpropanoids	99.90	Flavonoids	99.90	Flavanones	99.90
Taxifolin	Shikimates and Phenylpropanoids	99.90	Flavonoids	99.90	Dihydroflavonols	99.90
Quercetin 3-O-xylosyl-rutinoside	Shikimates and Phenylpropanoids	99.90	Flavonoids	99.90	Flavonols	99.90
Asebogenin	Shikimates and Phenylpropanoids	98.30	Flavonoids	99.90	Chalcones	99.90
Formononetin	Shikimates and Phenylpropanoids	99.90	Isoflavonoids	99.90	Isoflavones	99.90
calabricoside A	Shikimates and Phenylpropanoids	99.90	Flavonoids	99.90	Flavonols	99.90
tricin 7-O-($6^{\prime \prime }$-O-malonyl)-β-D-glucopyranoside	Shikimates and Phenylpropanoids	99.90	Flavonoids	99.90	Flavones	99.90
Acacetin	Shikimates and Phenylpropanoids	99.90	Flavonoids	99.90	Flavones	99.90
chrysoobtusin	Polyketides	99.60	Polycyclic aromatic polyketides	99.80	Anthraquinones and anthrones	98.30
1-AMINO-4-BENZAMIDO ANTHRAQUINONE	Polyketides	99.80	Polycyclic aromatic polyketides	99.90	Anthraquinones and anthrones	99.90
Quercetin 3-rutinoside-7-glucuronide	Shikimates and Phenylpropanoids	99.90	Flavonoids	99.90	Flavonols	99.90
Kaempferol 3-O-glucosyl-rhamnosyl-galactoside	Shikimates and Phenylpropanoids	99.90	Flavonoids	99.90	Flavonols	99.90
Syringetin-3-glucoside	Shikimates and Phenylpropanoids	99.90	Flavonoids	99.90	Flavonols	99.90
quercetin 3-O-(3-O-p-coumaroyl, 6-O-feruloyl)-glucoside	Shikimates and Phenylpropanoids	99.90	Flavonoids	100.00	Flavonols	99.90
1-Formyl-4-hydroxyanthraquinone	Polyketides	99.90	Polycyclic aromatic polyketides	99.90	Anthraquinones and anthrones	99.90
quercetin 3-O-gentiobioside-7-O-rhamnoside	Shikimates and Phenylpropanoids	99.90	Flavonoids	99.90	Flavonols	99.90
kaempferol 3-O-gentiobioside-7-O-rhamnoside	Shikimates and Phenylpropanoids	99.90	Flavonoids	99.90	Flavonols	99.90
Quercetin 3-glucosyl-(1→3)-rhamnosyl-(1→6)-galactoside	Shikimates and Phenylpropanoids	99.90	Flavonoids	99.90	Flavonols	99.90
Quercetin 3-rutinoside-7,$3^{\prime }$-diglucoside	Shikimates and Phenylpropanoids	99.90	Flavonoids	99.90	Flavonols	99.90
Ononin	Shikimates and Phenylpropanoids	99.90	Isoflavonoids	99.90	Isoflavones	99.90
Tetramethylscutellarein	Shikimates and Phenylpropanoids	99.90	Flavonoids	99.90	Flavones	99.90
Carnosol	Terpenoids	99.90	Diterpenoids	99.90	Abietane diterpenoids	99.90
KAEMPFEROL-3-O-($6^{\prime \prime \prime }$-TRANS-P-COUMAROYL-$2^{\prime \prime }$-GLUCOSYL)RHAMNOSIDE	Shikimates and Phenylpropanoids	99.90	Flavonoids	99.90	Flavonols	99.90
aromadendrin	Shikimates and Phenylpropanoids	99.90	Flavonoids	99.90	Dihydroflavonols	99.90
Biochanin A	Shikimates and Phenylpropanoids	99.70	Isoflavonoids	99.90	Isoflavones	99.80
Glycitein	Shikimates and Phenylpropanoids	99.90	Flavonoids	100.00	Flavones	99.90
(-)-L-Chicoric acid	Shikimates and Phenylpropanoids	97.30	Phenylpropanoids (C6–C3)	98.90	Cinnamic acids and derivatives	99.90
Scutellarin	Shikimates and Phenylpropanoids	99.90	Flavonoids	99.90	Flavones	99.90
Ellagic acid	Shikimates and Phenylpropanoids	99.90	Phenolic acids (C6–C1)	99.90	Gallotannins	99.90
Myricetin	Shikimates and Phenylpropanoids	99.80	Flavonoids	99.90	Flavonols	99.90
Luteolinidin	Shikimates and Phenylpropanoids	99.80	Flavonoids	99.90	Anthocyanidins	99.10
(E)-Ferulic acid	Shikimates and Phenylpropanoids	99.60	Phenylpropanoids (C6–C3)	99.10	Cinnamic acids and derivatives	99.20
quercetin 3-O-sophoroside-7-O-rhamnoside	Shikimates and Phenylpropanoids	99.90	Flavonoids	99.90	Flavonols	99.90
Glycitin	Shikimates and Phenylpropanoids	99.90	Isoflavonoids	99.90	Isoflavones	99.90
4-Heptyloxyphenol	Shikimates and Phenylpropanoids	24.30	Phenolic acids (C6–C1)	2.90	Hydrocarbons	3.00
$4^{\prime }$-HYDROXYFLAVONE	Polyketides	93.30	Monoterpenoids	13.10	Oblogolides	16.70
$2^{\prime \prime }$,$6^{\prime \prime }$-O-diacetyloninin	–		–		–	
ANTRAQUINONE DERIVATIVE	–		–		–	
6-(β-D-Glucopyranuronosyloxy)-5,7-dihydroxy-2-(4-hydroxyphenyl)-4-oxo-4H-chromen-8-yl β-D-glucopyranosiduronic acid	–		–		–	
2-(3,4-Dihydroxyphenyl)-7-(β-D-glucopyranosyloxy)-5-hydroxy-4-oxo-4H-chromen-3-yl 6-deoxy-α-L- mannopyranosyl-(1→2)-[6-deoxy-α-L-mannopyranosyl-(1→6)]-β-D-galactopyranoside	–		–		–	
5,7-Dihydroxy-2-(4-hydroxy-3-methoxyphenyl)-4-oxo-4H-chromen-3-yl β-D-glucopyranosyl-(1→3)-6-deoxy-α-L-mannopyranosyl-(1→6)-β-D-glucopyranoside	–		–		–	
Kaempferol 7-methyl ether 3- (6- (E) -3,5-dimethoxy-4-hydroxycinnamoylglucosyl) - (1→2) - [ rhamnosyl- (1→6) -glucoside ]	–		–		–	

**Table A10:** Discordant and unassigned superclass assignments in Rhubarb (8/83 compounds; overall concordance 90.0%).

Component	Validated Superclass	Predicted Superclass	Acc.%
4-Heptyloxyphenol	Aromatic polyketides	Phenolic acids (C6–C1)	2.9
$4^{\prime }$-HYDROXYFLAVONE	Flavonoids	Monoterpenoids	13.1
$2^{\prime \prime }$,$6^{\prime \prime }$-O-diacetyloninin	Flavonoids	–	
ANTRAQUINONE DERIVATIVE	Polycyclic aromatic polyketides	–	
6-(β-D-Glucopyranuronosyloxy)-5,7-dihydroxy-2-(4-hydroxyphenyl)-4-oxo-4H-chromen-8-yl β-D-glucopyranosiduronic acid	Flavonoids	–	
2-(3,4-Dihydroxyphenyl)-7-(β-D-glucopyranosyloxy)-5-hydroxy-4-oxo-4H-chromen-3-yl 6-deoxy-α-L-mannopyranosyl-(1→2)-[6-deoxy-α-L-mannopyranosyl-(1→6)]-β-D-galactopyranoside	Flavonoids	–	
5,7-Dihydroxy-2-(4-hydroxy-3-methoxyphenyl)-4-oxo-4H-chromen-3-yl β-D-glucopyranosyl-(1→3)-6-deoxy-α-L-mannopyranosyl-(1→6)-β-D-glucopyranoside	Flavonoids	–	
Kaempferol 7-methyl ether 3- (6- (E) -3,5-dimethoxy-4-hydroxycinnamoylglucosyl) - (1→2) - [ rhamnosyl- (1→6) -glucoside ]	Flavonoids	–	

The discordant and unassigned cases in Rhubarb mainly involve borderline or incompletely represented molecular structures. Misclassifications were associated with features that blur the boundaries between distinct chemical families, such as partial overlap between aromatic oxygenated systems and terpenoid-like motifs. The unassigned compounds correspond to highly glycosylated flavonoids and anthraquinone derivatives, for which complex substitution patterns extend beyond the canonical chemical space captured by the model. These instances highlight the model’s limitations in handling extensively conjugated or heavily substituted scaffolds, but were readily resolved through expert review and did not affect the conclusions at the superclass level.

### Appendix A.5. Senna

For Senna, Table A11 lists all identified metabolites with validated superclass assignments, while Table A12 provides the hierarchical classification with model-derived confidence scores. Table A13 highlights the few discordant cases, which are discussed further below. For full-precision *m*/*z* values (reported to four decimal places) together with the corresponding retention times (RT) and mass errors (Δ, ppm), readers are referred to Supplementary Table S1.

**Table A11:** Senna—Identified metabolites with validated *Superclass*. Molecular weights are rounded to two decimals.

Component	Formula	Molecular Weight	Superclass
vicenin 2	C_27_H_30_O_15_	594.16	Flavonoids
Rhein	C_15_H_8_O_6_	284.03	Polycyclic aromatic polyketides
Guaethol	C_8_H_10_O_2_	138.07	Phenylpropanoids (C6–C3)
1,2,4-Trihydroxyanthraquinone	C_14_H_8_O_5_	256.04	Polycyclic aromatic polyketides
Rhein-8-glucoside	C_21_H_18_O_11_	446.08	Polycyclic aromatic polyketides
11-o-Galloylbergenin	C_21_H_20_O_13_	480.09	Coumarins
Emodic acid	C_15_H_8_O_7_	300.03	Polycyclic aromatic polyketides
demethylwedelolactone	C_15_H_8_O_7_	300.03	Isoflavonoids
Sennoside A	C_42_H_38_O_20_	862.20	Isoflavonoids
Sennoside B	C_42_H_38_O_20_	862.20	Isoflavonoids
Sennidin B	C_30_H_18_O_10_	538.10	Isoflavonoids
Aloe emodin	C_15_H_10_O_5_	270.05	Isoflavonoids
Sennidin A	C_30_H_18_O_10_	538.10	Isoflavonoids
Luteolin	C_15_H_10_O_6_	286.05	Flavonoids
Eugenol	C_10_H_12_O_2_	164.08	Phenylpropanoids (C6–C3)
creosol	C_8_H_10_O_2_	138.07	Benzenoids
Myricetin	C_15_H_10_O_8_	318.04	Flavonoids
Coumesterol	C_15_H_8_O_5_	268.04	Isoflavonoids
Prodelphinidin T1	C_45_H_38_O_20_	898.20	Isoflavonoids
Quercitrin	C_21_H_20_O_11_	448.10	Isoflavonoids
Kaempferol	C_15_H_10_O_6_	286.05	Isoflavonoids
Rutin	C_27_H_30_O_16_	610.15	Isoflavonoids
(E)-Ferulic acid	C_10_H_10_O_4_	194.06	Isoflavonoids
Phloretin	C_15_H_14_O_5_	274.08	Flavonoids
Eriodictyol	C_15_H_12_O_6_	288.06	Flavonoids
Sinapinic acid	C_11_H_12_O_5_	224.07	Phenylpropanoids (C6–C3)
malonyldaidzin	C_24_H_22_O_12_	502.11	Isoflavonoids
Cinnamic acid	C_9_H_8_O_2_	148.05	Isoflavonoids
Daidzein	C_15_H_10_O_4_	254.06	Isoflavonoids
Cyanidin 3-O-sambubioside 5-O-glucoside	C_32_H_39_O_20_	743.20	Isoflavonoids
Carnosol	C_20_H_26_O_4_	330.18	Isoflavonoids
Irilone	C_16_H_10_O_6_	298.05	Isoflavonoids
olmelin	C_16_H_12_O_5_	284.07	Isoflavonoids
(±)-Naringenin	C_15_H_12_O_5_	272.07	Isoflavonoids
Peonidin 3-O-glucoside	C_22_H_23_O_11_	463.12	Isoflavonoids
Secoisolariciresinol	C_20_H_26_O_6_	362.17	Isoflavonoids
3,4-Dicaffeoylquinic acid	C_25_H_24_O_12_	516.13	Isoflavonoids
Sennoside C	C_42_H_40_O_19_	848.22	Polycyclic aromatic polyketides
Tectochrysin	C_16_H_12_O_4_	268.07	Flavonoids
Xanthorin	C_16_H_12_O_6_	300.06	Polycyclic aromatic polyketides
Miquelianin	C_21_H_18_O_13_	478.08	Flavonoids
Theaflavine	C_29_H_24_O_12_	564.13	Flavonoids
Rubrofusarin	C_15_H_12_O_5_	272.07	Naphthalenes
Procyanidin C1	C_45_H_38_O_18_	866.21	Flavonoids
Isorhamnetin 3-glucoside	C_22_H_22_O_12_	478.11	Flavonoids
(E)-p-coumaric acid	C_9_H_8_O_3_	164.05	Phenylpropanoids (C6–C3)
Hispidulin	C_16_H_12_O_6_	300.06	Flavonoids
4-Heptyloxyphenol	C_13_H_20_O_2_	208.15	Aromatic polyketides
$2^{\prime }$,$2^{\prime }$-BISEPIGALLOCATECHIN DIGALLATE	C_44_H_34_O_22_	914.15	Flavonoids
Diosmetin-O-glucoside	C_22_H_22_O_11_	462.12	Flavonoids
Quercetin 3-O-(2,6-di-O-rhamnosyl) galactoside	C_33_H_40_O_20_	756.21	Isoflavonoids

**Table A12:** Senna—Hierarchical classification of identified metabolites with model accuracy expressed as percentage.

Component	Pathway	Acc.%	Superclass	Acc.%	Class	Acc.%
vicenin 2	Shikimates and Phenylpropanoids	99.90	Flavonoids	99.90	Flavones	99.90
Rhein	Polyketides	99.90	Polycyclic aromatic polyketides	99.90	Anthraquinones and anthrones	99.90
Guaethol	Shikimates and Phenylpropanoids	66.30	Phenylpropanoids (C6–C3)	30.90	Cinnamic acids and derivatives	3.06
1,2,4-Trihydroxyanthraquinone	Polyketides	99.90	Polycyclic aromatic polyketides	99.90	Anthraquinones and anthrones	99.90
Rhein-8-glucoside	Polyketides	99.90	Polycyclic aromatic polyketides	99.90	Anthraquinones and anthrones	99.90
11-o-Galloylbergenin	Shikimates and Phenylpropanoids	96.60	Coumarins	96.40	Isocoumarins	95.10
Emodic acid	Polyketides	99.90	Polycyclic aromatic polyketides	99.90	Anthraquinones and anthrones	99.90
demethylwedelolactone	Shikimates and Phenylpropanoids	99.80	Isoflavonoids	98.70	Coumestan	99.90
Sennoside A	Polyketides	99.90	Polycyclic aromatic polyketides	99.90	Anthraquinones and anthrones	99.90
Sennoside B	Polyketides	99.90	Polycyclic aromatic polyketides	99.90	Anthraquinones and anthrones	99.90
Sennidin B	Polyketides	99.90	Polycyclic aromatic polyketides	99.90	Anthraquinones and anthrones	99.90
Aloe emodin	Polyketides	99.90	Polycyclic aromatic polyketides	99.90	Anthraquinones and anthrones	99.90
Sennidin A	Polyketides	99.90	Polycyclic aromatic polyketides	99.90	Anthraquinones and anthrones	99.90
Luteolin	Shikimates and Phenylpropanoids	99.90	Flavonoids	99.90	Flavones	99.90
Eugenol	Shikimates and Phenylpropanoids	99.90	Phenylpropanoids (C6–C3)	98.80	Cinnamic acids and derivatives	96.20
creosol	Shikimates and Phenylpropanoids	51.10	Phenylpropanoids (C6–C3)	11.00	Simple phenolic acids	22.90
Myricetin	Shikimates and Phenylpropanoids	99.80	Flavonoids	99.90	Flavonols	99.90
Coumesterol	Shikimates and Phenylpropanoids	99.90	Isoflavonoids	99.80	Coumestan	99.80
Prodelphinidin T1	Shikimates and Phenylpropanoids	100.00	Flavonoids	99.90	Proanthocyanins	99.90
Quercitrin	Shikimates and Phenylpropanoids	99.90	Flavonoids	99.90	Flavonols	99.90
Kaempferol	Shikimates and Phenylpropanoids	99.90	Flavonoids	99.90	Flavonols	99.90
Rutin	Shikimates and Phenylpropanoids	99.90	Flavonoids	99.90	Flavonols	99.90
(E)-Ferulic acid	Shikimates and Phenylpropanoids	99.60	Phenylpropanoids (C6–C3)	99.10	Cinnamic acids and derivatives	99.20
Phloretin	Shikimates and Phenylpropanoids	99.10	Flavonoids	99.90	Chalcones	99.90
Eriodictyol	Shikimates and Phenylpropanoids	99.90	Flavonoids	99.90	Flavanones	99.70
Sinapinic acid	Shikimates and Phenylpropanoids	99.70	Phenylpropanoids (C6–C3)	98.00	Cinnamic acids and derivatives	97.80
malonyldaidzin	Shikimates and Phenylpropanoids	99.90	Isoflavonoids	99.90	Isoflavones	99.90
Cinnamic acid	Shikimates and Phenylpropanoids	99.00	Phenylpropanoids (C6–C3)	95.20	Cinnamic acids and derivatives	91.70
Daidzein	Shikimates and Phenylpropanoids	99.90	Isoflavonoids	99.90	Isoflavones	99.90
Cyanidin 3-O-sambubioside 5-O-glucoside	Shikimates and Phenylpropanoids	99.90	Flavonoids	99.90	Anthocyanidins	99.90
Carnosol	Terpenoids	99.90	Diterpenoids	99.90	Abietane diterpenoids	99.90
Irilone	Shikimates and Phenylpropanoids	99.90	Isoflavonoids	99.90	Isoflavones	99.70
olmelin	Shikimates and Phenylpropanoids	99.70	Isoflavonoids	99.90	Isoflavones	99.80
(±)-Naringenin	Shikimates and Phenylpropanoids	99.90	Flavonoids	99.90	Flavones	99.90
Peonidin 3-O-glucoside	Shikimates and Phenylpropanoids	99.90	Flavonoids	99.90	Anthocyanidins	99.90
Secoisolariciresinol	Shikimates and Phenylpropanoids	99.90	Lignans	100.00	Dibenzylbutane lignans	99.90
3,4-Dicaffeoylquinic acid	Shikimates and Phenylpropanoids	99.30	Phenylpropanoids (C6–C3)	99.40	Cinnamic acids and derivatives	99.90
Sennoside C	Polyketides	99.90	Polycyclic aromatic polyketides	99.90	Anthraquinones and anthrones	99.90
Tectochrysin	Shikimates and Phenylpropanoids	99.30	Flavonoids	99.90	Flavones	99.90
Xanthorin	Polyketides	99.90	Polycyclic aromatic polyketides	99.90	Anthraquinones and anthrones	99.90
Miquelianin	Shikimates and Phenylpropanoids	99.90	Flavonoids	99.90	Flavonols	99.90
Theaflavine	Shikimates and Phenylpropanoids	99.90	Flavonoids	99.90	Flavan-3-ols	99.90
Rubrofusarin	Polyketides	99.60	Naphthalenes	77.90	Naphthalenes and derivatives	98.30
Procyanidin C1	Shikimates and Phenylpropanoids	100.00	Flavonoids	99.90	Proanthocyanins	99.90
Isorhamnetin 3-glucoside	Shikimates and Phenylpropanoids	99.90	Flavonoids	99.90	Flavonols	99.90
(E)-p-coumaric acid	Shikimates and Phenylpropanoids	99.00	Phenylpropanoids (C6–C3)	95.10	Cinnamic acids and derivatives	97.50
Hispidulin	Shikimates and Phenylpropanoids	99.90	Flavonoids	99.90	Flavones	99.90
4-Heptyloxyphenol	Shikimates and Phenylpropanoids	24.30	Phenolic acids (C6–C1)	2.90	Hydrocarbons	3.10
$2^{\prime }$,$2^{\prime }$-BISEPIGALLOCATECHIN DIGALLATE	Shikimates and Phenylpropanoids	99.90	Phenolic acids (C6–C1)	99.70	Flavan-3-ols	94.60
Diosmetin-O-glucoside	Shikimates and Phenylpropanoids	99.70	Coumarins	91.40	Furocoumarins	21.60
Quercetin 3-O-(2,6-di-O-rhamnosyl) galactoside	–		–		–	

**Table A13:** Discordant and unassigned superclass assignments in Senna (4/51 compounds; overall concordance 92.3%).

Component	Validated Superclass	Predicted Superclass	Acc.%
4-Heptyloxyphenol	Aromatic polyketides	Phenolic acids (C6–C1)	2.9
$2^{\prime }$,$2^{\prime }$-BISEPIGALLOCATECHIN DIGALLATE	Flavonoids	Phenolic acids (C6–C1)	99.7
Diosmetin-O-glucoside	Flavonoids	Coumarins	91.4
Quercetin 3-O-(2,6-di-O-rhamnosyl) galactoside	Isoflavonoids	–	

The discordant and unassigned cases in Senna mainly concern polyphenolic structures with overlapping functional motifs. The misclassification of large flavonoid derivatives such as $2^{\prime }$,$2^{\prime }$-bisepigallocatechin digallate and diosmetin-O-glucoside reflects partial similarity with phenolic or coumarin-like scaffolds that share conjugated aromatic systems and hydroxylation patterns. The unassigned compounds correspond to highly glycosylated anthraquinone and isoflavonoid derivatives, for which the model could not confidently assign a superclass due to the structural complexity and rare substitution patterns. Overall, these few discrepancies do not affect the reliability of the classification at the superclass level, confirming the robustness of the annotation workflow.
